# SOX2 promotes resistance of melanoma with PD-L1 high expression to T-cell-mediated cytotoxicity that can be reversed by SAHA

**DOI:** 10.1136/jitc-2020-001037

**Published:** 2020-11-06

**Authors:** Ruiyan Wu, Caiqin Wang, Zhiming Li, Jian Xiao, Chunyan Li, Xuemin Wang, Pengfei Kong, Jianghua Cao, Fuxue Huang, Zhiling Li, Yun Huang, Yuhong Chen, Xuan Li, Dong Yang, Hailiang Zhang, Jia Mai, Gongkan Feng, Rong Deng, Xiaofeng Zhu

**Affiliations:** 1 State Key Laboratory of Oncology in South China, Collaborative Innovation Center for Cancer Medicine, Guangdong Key Laboratory of Nasopharyngeal Carcinoma Diagnosis and Therapy, Sun Yat-sen University Cancer Center, Guangzhou, China; 2 Department of Radiation Oncology, Fudan University Shanghai Cancer Center, Shanghai, China; 3 Department of Medical Oncology, Sun Yat-sen University Sixth Affiliated Hospital, Guangzhou, Guangdong, China; 4 Department of Medical Oncology, Sun Yat-sen University Cancer Center, Guangzhou, Guangdong, China; 5 Department of The Second Head and Neck Surgery, The Third Affiliated Hospital of Kunming Medical University, Kunming, Yunnan, China; 6 Department of Surgery, Fudan University Shanghai Cancer Center, Shanghai, Shanghai, China

**Keywords:** CD8-positive T-lymphocytes, immunotherapy, melanoma, tumor escape, drug therapy, combination

## Abstract

**Background:**

Immune checkpoint inhibitors (ICIs) induce better tumor regression in melanoma with programmed cell death 1 ligand 1 (PD-L1) high expression, but there has been an upsurge of failed responses. In this study, we aimed to explore the additional mechanisms possibly accounting for ICIs resistance and interventional strategies to overcome the resistance in melanoma with PD-L1 high expression.

**Methods:**

Melanoma xenografts and cytotoxicity assays were used to investigate function of SOX2 in regulating antitumor immunity. The activity of the janus kinase-signal transducer and activator of transcriptions (JAK-STAT) pathway was investigated by western blots, quantitative PCR and luciferase assay. Epigenetic compounds library screen was employed to identify inhibitors that could decrease SOX2 level. The effect of histone deacetylase inhibitor SAHA in antitumor immunity alone or in combination with immunotherapy was also determined in vitro and in vivo. Prognostic impact of SOX2 was analyzed using transcriptional profiles and clinical data download from the Gene Expression Omnibus and The Cancer Genome Atlas repository.

**Results:**

We uncovered a role of SOX2 in attenuating the sensitivity of melanoma cells to CD8+ T-cell killing. Mechanistically, SOX2 inhibited phosphatases suppressor of cytokine signaling 3 (SOCS3) and protein tyrosine phosphatase non-receptor type 1 (PTPN1) transcription, induced duration activation of the JAK-STAT pathway and thereby overexpression of interferon stimulated genes resistance signature (ISG.RS). By targeting the SOX2-JAK-STAT signaling, SAHA promoted the antitumor efficacy of IFNγ or anti-PD-1 in vitro and in vivo. Moreover, SOX2 was an independent prognostic factor for poor survival and resistant to anti-PD-1 therapy in melanoma with PD-L1 high expression.

**Conclusions:**

Our data unveiled an additional function of SOX2 causing immune evasion of CD8+ T-cell killing through alleviating the JAK-STAT pathway and ISG.RS expression. We also provided a rationale to explore a novel combination of ICIs with SAHA clinically, especially in melanoma with PD-L1 and SOX2 high expression.

## Background

Immune checkpoint inhibitors (ICIs) targeting the cytotoxic T lymphocyte antigen 4 (CTLA-4), programmed death 1 (PD-1) and programmed cell death 1 ligand 1 (PD-L1) have elicited durable tumor control in patients with various cancer types.^1 2^ However, the sustained benefit has been observed in a small proportion of patients with a response rate ranging from 10% to 35%.^3^ Immunogenic tumors, characteristic with T cells and T cell-inflamed gene expression profile (CD8, IFNγ, GranzymeB, PD-L1), are associated with favor survival and response to ICIs.^4^ Metastatic melanoma are highly immunogenic with a relatively high mutational landscape, T-cell infiltrating and PD-L1 expression.^5^ According to previous studies, about 80% metastatic melanoma with PD-L1 expression >1% in tumor cells and 25% patients with PD-L1 expression >5% in tumor cells.^6 7^ It makes ICIs as promising treatment options for metastatic melanoma. Despite successes, thus far, the overall response rate was 57% in PD-L1 positive patients.^8^ It is becoming a pressing necessity to identify additional biomarker predicting respond to ICIs in melanoma with PD-L1 expression and clarify the mechanism.

SOX2 is a transcription factor known for its essential role in stem cell biology, cellular differentiation and tumorigenesis.^9^ Recent studies have observed controversial function of SOX2 in regulating antitumor immunity. SOX2 was reported as a tumor suppressor: a sequence-specific DNA sensor in neutrophils to initiate innate immunity^10^ or a tumor-associated antigen recognized by specific CD4+ and CD8+ T cell to activate adaptive immunity.^11 12^ Contrarily, some reports have revealed oncogenic functions of SOX2 in eliciting autophagy-dependent degradation of stimulator of interferon genes (STING) to impair innate immunity^13^ or through transcription activating PD-L1 expression to cause adaptive immune resistance.^14^ To date, the biological role of SOX2 has not been clearly established, especially in regard to reciprocal interactions between tumor cells and host antitumor immune response.

In this study, we found that SOX2 caused resistant to T-cell-mediated cytotoxicity and anti-PD-1 in melanoma with PD-L1 high expression through inducing overexpression of ISG.RS. Based on epigenetic compounds library screen, we identified that SAHA is one of the promising agents in enhancing the effect of anti-PD-1 by targeting SOX2. Our data indicated a new paradigm in which tumor immunogenicity was regulated through SOX2 in tumor cells, leading to possible implications for targeting SOX2 in cancer immunotherapy.

## Materials and methods

### Cell culture

Melanoma MM200, ME4405, MEL-RM and B16/F10 were purchased from the American Type Culture Collection (ATCC, Manassas, VA, USA). The obtained cell lines were authenticated by short tandem repeat (STR) profiling through the Victorian Centre for Functional Genomics. The most recent authentication was on March 1, 2017. The cells were maintained in a recommended medium with 10% fetal bovine serum (Gibco, Grand Island, NY, USA) and antibiotics (50 mg/mL-1penicillin/streptomycin) and routinely tested for Mycoplasma species prior to performing related experiments.

### Plasmids and siRNAs

The plasmids encoding human or mouse SOX2 (pmCherry-Flag-hSOX2 and pCDH-Flag-mSOX2), plasmid knockdown (KD) human SOX2 (pLKO.1-hSOX2) and plasmid knockout mouse SOX2 (pLenti-V2-mSOX2) were constructed, and siRNAs were obtained from GenePharma (Shanghai, CHN). Transfection of plasmids or siRNAs to melanoma cells was performed using the Lipofectamine 2000 (No. 11668019, Invitrogen, Carlsbad, CA, USA) or Lipofectamine RNAiMAX transfection reagent (No. 13 778–150, Invitrogen, Carlsbad, CA, USA) according to the manufacturer’s instructions. Information for primer sequences are as listed: siSOX2#1/shSOX2#1, human, 5′-AGAAATATCCTCCTTACTC-3′; siSOX2#2/shSOX2#2, human, 5′-TGACGTCAATGCTGCCATA-3′, siSOX2#3, human, 5′CGAGGUGCUGAGCAAGAAA-3′, sgSOX2#1, mouse, 5′-GCAGGGCGCTGACGTCGTAG-3′, sgSOX2#2, mouse, 5′-CATGTATAACATGATGGAGA-3′.

### Antibodies and reagents

The antibodies used for western blotting: anti-SOX2 (No. ab92494, abcom, Cambridge, MA, USA), anti-p-JAK1 (Tyr1022/1023) (No. GTX50207, GeneTex, San Antonio, TX, USA), anti-JAK2 (No. GTX31943, GeneTex), anti- SOCS3 (No. A0694, Abclonal, Wuhan, CHN), anti-PTPN1 (No. A1590, Abclonal), anti-GAPDH (No. 60 004–1, Proteintech, Chicago, IL, USA). Anti-JAK1 (No. 3344S), anti-p-JAK2 (Tyr1007/1008) (No. 3776S), anti-STAT1 (No. 9172S), anti-p-STAT1 (Tyr701) (No. 7649S), anti-STAT3 (No. 9139S), anti-p-STAT3 (Tyr705) (No. 9145S), anti-Acetylated-Lysine (No. 9681S) and anti-β-Tubulin (No. 2146S) were purchased from Cell Signaling Technology (Danvers, MA, USA).

Flow cytometry antibodies: Alexa Fluor 488 antimouse CD3ε (No. 100321), Alexa Fluor 647 antihuman/mouse GranzymeB (No. 515406) and PerCP/Cyanine5.5 antimouse IFNγ (No. 505821) were purchased from Biolegend (San Diego, CA, USA). Ms CD4 PE-Cy7 RM4-5 (No. 552775), Ms CD8a PerCP-Cy5.5 53–6.7 (No. 551162), FVS700 (No. 564997), Ms CD45.1 PerCP-Cy5.5 A20 (No. 560580), Ms Foxp3 Alexa 647 R16-715 (No. 563486) and PE antimouse NK cells (NK1.1) (No. 557391) were purchased from BD Pharmingen (San Diego, CA, USA).

The epigenetic compounds library, inhibitors and cytokines are as listed: epigenetic compounds library (No. L1900, Selleck Chemicals, Houston, TX, USA), SAHA (No. S1047, Selleck Chemicals), Etidronate (No. S1857, Selleck Chemicals), PTP1B inhibitor (No. T4256, TargetMol, Boston, MA, USA), protein tyrosine phosphatase (PTP) inhibitor I (No. T7084, TargetMol), SPI 112 (No. T4340, TargetMol), murine IFNγ (No. 315-05-100, PeproTech, Rocky Hill, NJ, USA), human IFNγ (No. 300-02-1000, PeproTech), human IL2 (No. 200-02-100, PeproTech), antimouse PD-1 (CD279) (No. BE0146, BioXCell, West Lebanon, NH, USA), Rat IgG2a isotype control (No. BE0089, BioXCell).

### Mice

Six-week-old C57BL/6 and nude mice were procured from the Laboratory Animal Center of Guangdong province (Guangzhou, CHN). These animals were maintained in accordance with the institutional guidelines and the experiments were approved by the Animal Care and Use Committee of Sun Yat-sen University Cancer Center.

### Animal experiments

5×10^6^ human MEL-RM cells were subcutaneously injected into the flank of nude mice or 0.5×10^6^ B16/F10 cells were injected into the back flank C57BL/6 mice. Four days following the injection, the mice were randomized into the following four groups: (1) vehicle; (2) treatment with SAHA (25 mg/kg, intraperitoneally, every other days); (3) treatment with anti-PD-1 (100 µg per mice, intraperitoneally, every 3 days) or IFNγ (2.5 µg per mice, intraperitoneally, every 3 days); (4) treatment with a combination of SAHA and anti-PD-1 or IFNγ. The tumors were measured every third-day for nude mice or second-day for CB7BL/6 mice and tumor volume was calculated as (length x width^2^)/2. Tumor growth was monitored over a period of 8 days and the mice were sacrificed.

### Flow cytometry

The tumors were cut into small pieces, mechanically disrupted and filtered through a 70 µm mesh to generate a single-cell suspension. Dissociated tumor cells were lysed with Red Blood Cell Lysis Solution (No. 420301, Biolegend) and incubated with phorbol 12-myristate 13-acetate (PMA) (No. P1585, Sigma-Aldrich, St. Louis, MO, USA), ionomycin (No. 73724, Stemcell, Vancouver, BC, CAN) and Golgi stop (No. 554715, BD Pharmingen) at 37°C. For cell surface staining, cell suspensions were stained with indicated fluorescent labeled antibodies for 30 min on ice. For intracellular staining, the cells were sorted for fixation and permeabilization using Cytofix/CytoPerm BUF KIT (No. 554714, BD Pharmingen) according to the manufacturer’s guidelines and incubated with primary antibodies. Flow cytometry acquisition was carried out on LSRFortessa (BD Biosciences, San Jose, CA, USA) or a Navios and Gallios (Beckman Coulter, Miami, FL, USA). Compensation beads were used to evaluate spectral overlap. The data were analyzed using FlowJo and CytExpert software according to manufacturers’ instructions.

### Cytotoxicity assays in vitro

CD3+ T cells were isolated from human peripheral blood mononuclear cells and activated with 10 ug/mL LEAF Purified anti-CD3 (No. 300314, Biolegend), 2 ug/mL LEAF Purified anti-CD28 (No. 302913, Biolegend) and 100 IU/mL human IL2 (No. 200-02-100, PeproTech) as previously reported.^15^ Pretreated melanoma cells were cocultured with the activated T cell in a ratio of 10:1 or 20:1 for 6–8 hours. The cells were sorted, stained with FITC Anti-Active Caspase-3 (No. 559341, BD Pharmingen) and used for flow cytometric analysis.

### Cell proliferation analysis

For colony formation assay, a total of 300 melanoam cells were seeded in a 6-well plate and cultured in complete DMEM medium for 10 days. Colonies were fixed with 4% polymethanol and dyed with 0.1% crystal violet (1 mg/mL), and the number of colonies with over 50 cells was counted. For CCK-8 assay, a total of 300 melanoam cells were seeded in triplicate in each well of a 96-well plate, and the cell numbers were counted every day by CCK-8 (No. CK04-500, DOJINDO, Kumamoto, Japan) for 7 days.

### Western blot

The cells were lysed in 1x Cell Lysis Buffer (No. 9873, CST) supplemented with protease inhibitor phenylmethylsulphonyl fluoride. Protein was quantitated using the BCA Protein Assay Kit (No. 23227, Thermo Fisher Scientific, Waltham, MA, USA). Equal amounts of protein were boiled by adding 6x SDS sample buffer for 10 min at 100°C and resolved using SDS-PAGE. Membranes were blocked in 5% milk/TBS-T (Boston Bioproducts) and incubated with their respective antibodies.

### RNA isolation and quantitative PCR (qPCR)

Total RNA was extracted using TRIzol reagent (No. 15596018, Invitrogen) according to the manufacturer’s instructions. The RNA concentration was determined using a Nanodrop (Thermo Fisher Scientific). cDNA was reversed from 1 µg of total RNA using the HiScript II Q Reverse Transcription kit (No. R233-01, Vazyme, Nanjing, CHN). QPCR amplification was performed using the SYBR master mix plus (No. Q311-03, Vazyme) and run on Bio-Rad CFX96 qPCR system. All reactions were performed in duplicate. Relative gene expression was quantified and normalized to GAPDH. Both the forward and reverse primers used are designed form Primerbank https://pga.mgh.harvard.edu/primerbank.

### Luciferase assay

HEK293T cells were cotransfected with luciferase-reporter plasmids and PGL3 or PGL3-SOCS3/PGL3-PTPN1 plasmids using the Lipofectamine 2000 transfection reagent. Lysis buffer (25 mM of Tris phosphate, 1% Triton X-100, 1 mM of dithiothreitol, 2 mM of ethylenediaminetetraacetic acid, 10% glycerol, PH=7.8) was added at 48 hours post-transfection. Luciferase assay was performed using a luciferase assay kit (No. E1910, Promega, Madison, WI, USA). An enzyme-linked immunosorbent-assay plate reader (Bio-Rad) was applied to measure O-nitrophenol at a wavelength of 490 nm to evaluate β-galactosidase activity.

### Epigenetic compound library screen

Epigenetic compound library was purchased from Selleck. Five hundred thousand MM200 cells were seeded overnight in 12-well plates. Following IFNγ stimulation, the inhibitors were added to the culture medium at a concentration of 10 µM. After 24 hours, the cells were harvested and lysed for western blot.

### Human clinical and gene expression data

Normalized gene expression data and paired clinical feature data of patients without immunotherapy were download from the The Cancer Genome Atlas (TCGA) database (https://tcga-data.nci.nih.gov). Data of patients treated with nivolumab (accession number GSE 91061^16^) or pembrolizumab (accession number GSE78220^17^) were obtained from Gene Expression Omnibus (GEO) database (http://www.ncbi.nlm.nih.gov/geo). Overall survival (OS) was defined as the time from randomization to death from any cause and progression-free survival (PFS) was defined as the time from randomization to documented disease progression according to Response Evaluation Criteria in Solid Tumors, version 1.1 (RECIST V.1.1) or death from any cause. Objective response (ORR) was the proportion of patients with confirmed complete response or partial response according to RECIST V.1.1.^18^

The optimal cut-off for PD-L1 was the median, meaning that mRNA level ranking at the top 50% of the patients was defined as PD-L1 high and the others were PD-L1 low.

The optimal cut-offs for SOX2 were determined using receiver operating characteristic (ROC) analysis. In the prenivolumab cohort (GSE96061), the cut-off of SOX2 was 0.054, the sensitivity, specificity and area under the curve (AUC) were 77.8, 87.5% and 79.7%, respectively (p=0.017). In the onnivolumab cohort (GSE96061), the cut-off of SOX2 was 0.204, the sensitivity, specificity and AUC were 80.0, 80.0% and 80.0%, respectively (p=0.013). In the perpembrolizumab cohort (GSE96061), the cut-off of SOX2 was 0.925, the sensitivity and specificity were 83.3% and 57.1%, respectively.

### Statistical analysis

Kaplan-Meier plots for OS and PFS were compared using the log-rank test. The comparison of clinicopathological characteristics was performed with the use of a log-rank test stratified at a two-sided alpha level. A Cox proportional-hazards model was used to estimate the HR and its corresponding CI for death. The R programming language (http://cran.r-project.org) was used to classify the different gene signature patterns of the patients. The DAVID database (Database for Annotation, Visualization and Integration Discovery, http://david.abcc.ncifcrf.gov) was used to conduct a functional enrichment analysis.^19^ Statistical analyses were performed using GraphPad Prism (V.5.0, http://www.graphpad.com) and SPSS V.24.0. All data were presented as the mean±SD or were otherwise noted in the legends. All reported p values were two-tailed. For all analyses, p<0.05 was considered as statistically significant unless specified otherwise. All data shown are representative of two or more independent experiments, unless indicated otherwise.

## Results

### SOX2 caused immune evasion of CD8+ T-cell cytotoxic effect

**Figure 1:**
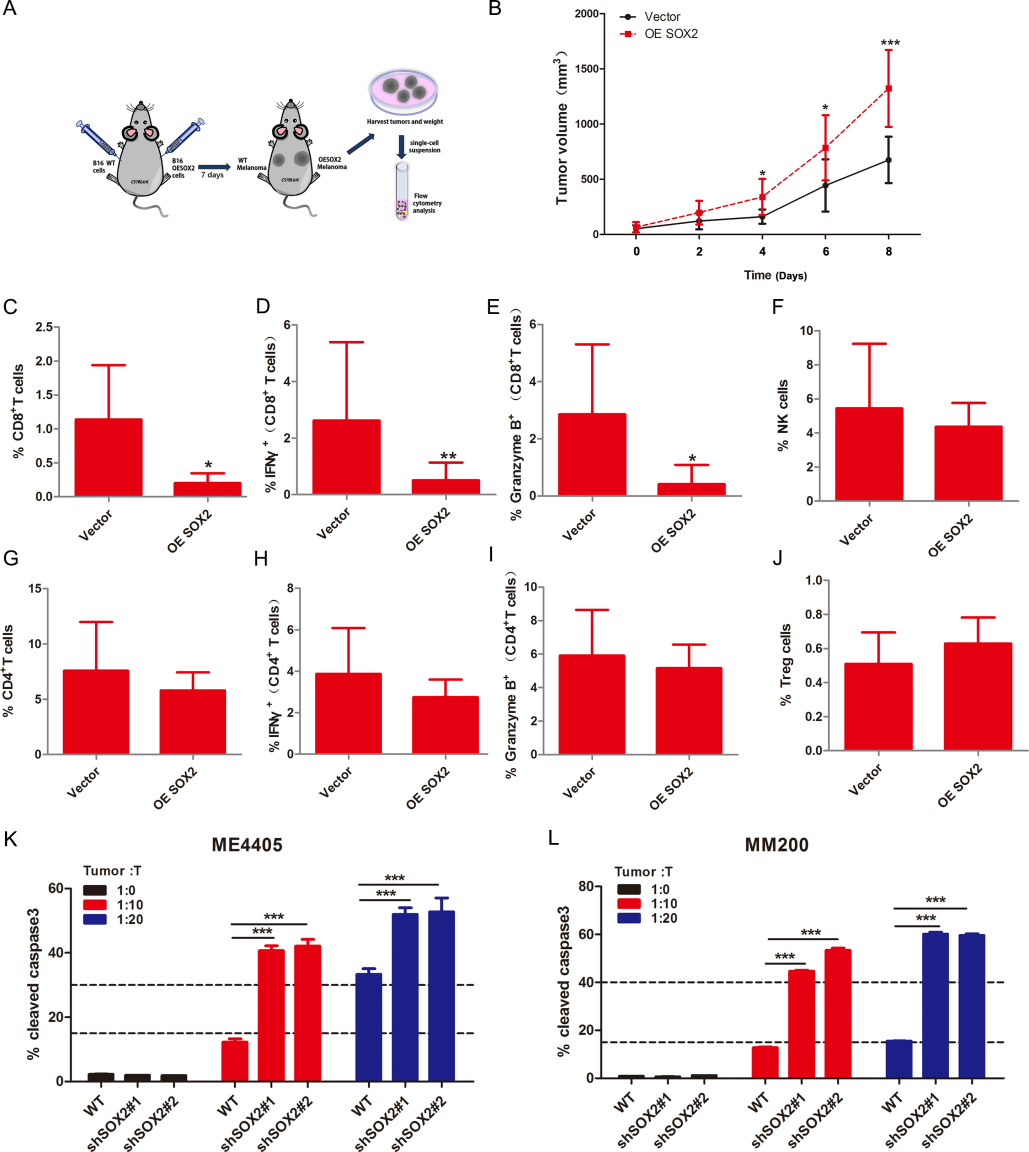
SOX2 attenuated CD8+ T-cell killing in vivo and in vitro. (A) The in vivo experimental layout. (B) Tumor growth curves of melanoma tumors from C57BL/6 mice (B16/F10 vector or OE-SOX2 group, n=6). (C, G) Percentage of CD8+ T (indicated by CD3+ CD8+ staining) and CD4+ T (indicated by CD3+ CD4+ staining) in tumor infiltrating lymphocytes (TILs) for each group. (D, E, H, I) Percentage of cytotoxic CD8+ T in total CD8+ T cells and cytotoxic CD4+ T in total CD4+ T cells. The cytotoxic T cells were indicated by intracellular staining of IFNγ or GranzymeB. (F) Percentage of NK cells (indicated by CD3+ NK1.1+ staining) in TILs. (J) Percentage of Treg cells (indicated by CD25+ and FOXP3+ costaining) in TILs. (K, L) Histogram of the percentage of cleaved caspase-3 positive cells assessed by flow cytometry. Vector and SOX2 OE melanoma cells were cocultured with T cells (Tumor: T=10:1 or 20: 1). Tumor: T, Tumor cells: T cells. Error bars indicate SEM. Unpaired two-tailed t tests for two groups comparison. One-way ANOVA test for three or more groups comparison. ANOVA, analysis of variance; NS, not significant; ^***^ p<0.001, ^**^p<0.01, ^*^p<0.05.

To establish relationship between SOX2 and host antitumor immune response in vivo, vector and SOX2 OE B16/F10 cell lines were generated and verified by western blot (online supplemental figure 1A) and transplanted into the syngeneic C57BL/6 mice (figure 1A). SOX2 OE promoted melanoma growth as confirmed by the growth curve of the xenografts volume (figure 1B). Immune cell subsets pooled from tumor infiltrating lymphocytes were analyzed by multicolor flow cytometry analysis (online supplemental figure 1B). The results showed that SOX2 OE suppressed CD8+ T cells (figure 1C, online supplemental figure 1C), while had no influence on CD4+ T/effector T cells, natural killer (NK) cells and Treg cells (figure 1F–J). Importantly, CD8+ T cells infiltration were accompanied by a decreasing in cytotoxic function, which was confirmed by GranzymeB and IFNγ staining (figure 1D and E, online supplemental figure 1C). Next, we investigated whether SOX2 could impair CD8+ T-cell killing in vitro. We generated SOX2 KD melanoma cell lines (ME4405 and MM200) by indicated shRNA (online supplemental figure 2A, B) and performed a T-cell-mediated cytotoxicity assays. SOX2 KD enhanced the sensitivity of melanoma cells to T-cell killing (figure 1K and L). We next validated whether SOX2 exhibited direct impact on tumor growth in vitro. As assessed by colony formation and CCK8 assay, we found SOX2 OE (online supplemental figure 2C) or KD had mild effect on colony formation (online supplemental figure 2D, E) and tumor growth (online supplemental figure 2F–I). These finding suggested that SOX2-related tumor growth was caused by immune evasion of CD8+ T-cell killing.

10.1136/jitc-2020-001037.supp1 Supplementary data

To explore the impact of SOX2 on treatment-naive patients OS, 420 melanoma patients data download from TCGA database was analyzed. The result showed that SOX2 high status indicated a poor OS (p value, 0.031) in CD8+ T-cell proficient patients while it could not stratify survival in CD8+ T-cell absent patients (online supplemental figure 2J). IFNγ and GranzymeB were both effectors of cytotoxic T cell and had a critical role in antitumor immunity, we also observed that SOX2 predicted poor OS in IFNγ or GranzymeB high expression patients (p value, IFNγ: 0.016, GranzymeB: 0.031). Besides, IFNγ or GranzymeB low expression could not contribute to the apparent discrepancy of OS between different SOX2 statuses patients (online supplemental figure 2K, L). Together, we concluded that SOX2 prompted tumor progression through causing CD8+ T-cell tolerance.

### SOX2 promoted the JAK-STAT pathway activity and ISG.RS expression

**Figure 2:**
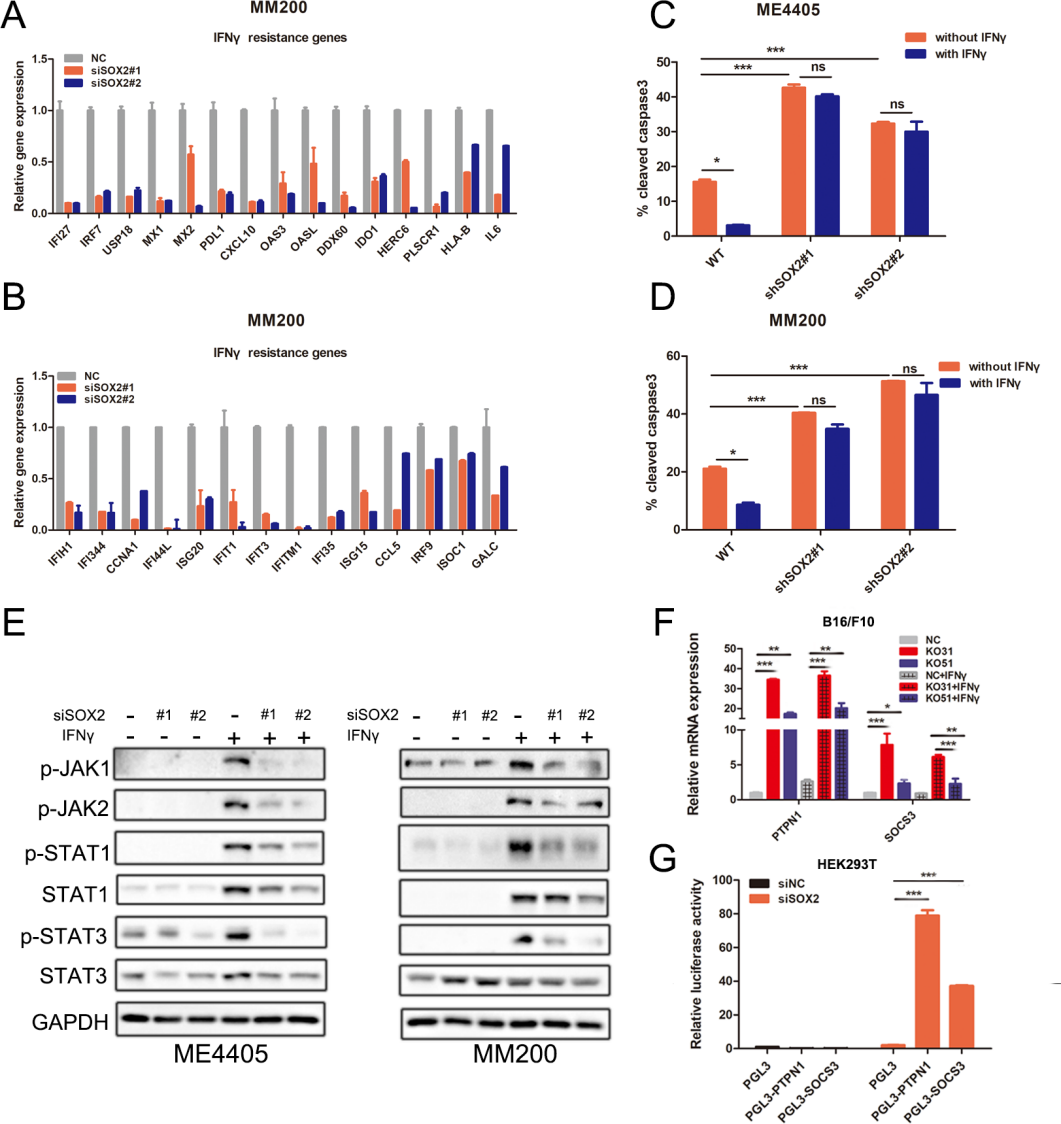
SOX2 increased JAK-STAT pathway activity and ISG.RS expression. (A, B) The expression of ISG.RS mRNA assessed by qPCR. MM200 cells were transiently transfected with indicated siRNA for 48 hours. Genes were shown for transcripts that were decreased more than twofold. (C, D) Histogram of the percentage of cleaved caspase-3 positive cells among each group. ME4405 and MM200 cells were transfected with indicated shRNA, treated with or without 1000 IU/mL IFNγ for 24 hours and then cocultured with T cells for 6–8 hours (tumor: T=10:1). (E) Change of the p-JAK1/2, p-STAT1/3 level determined by Western blot. ME4405 and MM200 cells were transfected with indicated siRNA for 24 hours and then treated with or without 1000 IU/mL IFNγ for another 24 hours. (F) Change of PTNP1 and SOCS3 mRNA determined by qPCR. SOX2 knock-out B16/F10 cell clones were treated with or without 1000 IU/mL IFNγ for 24 hours. (G) The transcription of PTNP1 and SOCS3 assessed by luciferase report assay. HEK293T cells were transiently transfected with indicated siRNA. Tumor: T, Tumor cells: T cells. Error bars indicate SEM. One-way ANOVA test. ANOVA, analysis of variance; NS, not significant; SOCS3, suppressor of cytokine signaling; ^***^p<0.001, ^**^p<0.01, ^*^p<0.05.

Since the duration of tumor IFNγ signaling and overexpression of ISG.RS accounted for the resistant to ICIs,^20 21^ we hypothesized that SOX2 impaired CD8+ T-cell killing through regulating the IFNγ signaling. We detected ISG.RS mRNA level in negative control or SOX2 KD melanoma cell lines and observed that SOX2 KD decreased the ISG.RS expression (IDO1, PDL1, IFI27, and USP16) (figure 2A, B). ME4405 and MM200 cells were pretreated with IFNγ to induce ISG.RS expression and then cocultured with activated human peripheral blood T cells. We found IFNγ exposures caused melanoma cells escaping from T-cell killing. When SOX2 was knocked down, the sensitivity was recovered without significantly difference between the IFNγ treated and untreated group (figure 2C, D), speculating that SOX2 KD regained the sensitivity to T-cell killing depending on attenuating IFNγ-induced ISG.RS expression.

Next, we further explored how the transcriptional repressor SOX2 regulated ISG.RS expression. We found SOX2 KD suppressed the JAK-STAT pathway activity (figure 2E). Previously reported that suppressor of cytokine signaling family and protein tyrosine phosphatase family coud counter the activities of JAK-STATs pathway,^22 23^ we proposed that SOX2 inhibition might increase phosphatase expression. In fact, we found that SOX2 knockout (online supplemental figure 2M) increased SOCS3 and PTPN1 mRNA level (figure 2F). Further, luciferase report assay confirmed that SOCS3 and PTPN1 were transcriptionally induced by SOX2 KD (figure 2G). Together, SOX2 caused immune evasion to CD8+ T-cell killing through inhibiting SOCS3 and PTPN1 transcription, further inducing the hyperactivation of the JAK-STAT pathway and OE of ISG.RS.

### Epigenetic compounds library screen identified SAHA decreasing SOX2 level

**Figure 3:**
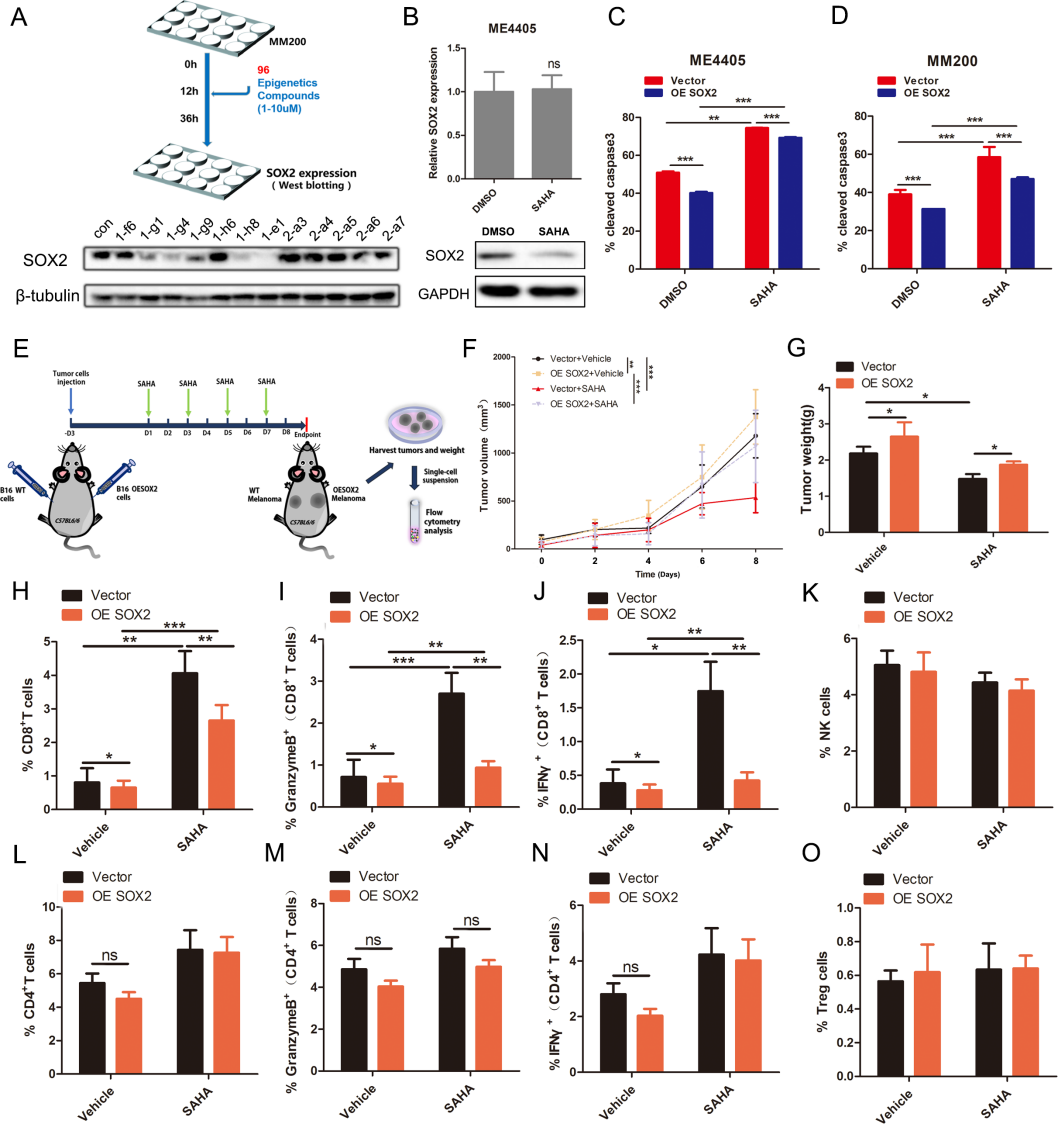
Epigenetic compounds library screen identified SAHA decreased SOX2 level and eliminated SOX2-dependent tumor growth. (A) Schematic illustration of the screen strategy. MM200 cells were treated with 1000 IU/mL IFNγ and 5 µM epigenetic inhibitors for 24 hours, then the protein level of SOX2 was assessed by western blot assay. (B) Change of SOX2 protein level and mRNA according to Western blot and qPCR assay. ME4405 cells were treated with or without 5 µM SAHA for 24 hours. (C, D) Histogram of the percentage of cleaved caspase-3 positive cells assessed by flow cytometry. Vector and SOX2 OE ME4405 and MM200 cells were treated with or without 5 µM SAHA for 24 hours and then cocultured with T cells for 6–8 hours (Tumor: T=10:1). (E) The in vivo experimental layout. (F, G) Tumor growth curve and xenografts weight of melanoma tumors from C57BL/6 mice (control and SAHA, n=8). (H, K, L, O) Percentage of CD8+ T, NK, CD4+ T and Treg T cells in TILs for each group. (I, J, M, N) Percentage cytotoxic CD8+ T in total CD8+ T cells and cytotoxic CD4+ T in total CD4+ T cells. Tumor: T, Tumor cells: T cells. Error bars indicate SEM. One-way ANOVA test. NS, not significant; TILs, tumor infiltrating lymphocytes; ^***^p<0.001, ^**^p<0.01, ^*^p<0.05.

Since SOX2 caused immune evasion of CD8+ T-cell killing, we assumed that suppressing SOX2 could argument antitumor immunity. As methylation and acetylation promoted SOX2 ubiquitination and proteasome degradation,^24 25^ we performed western blot-based screen using an epigenetic compound library to identify epigenetic inhibitors decreasing SOX2. A collection of 96 agents were tested and 20 histone deacetylases could decrease the SOX2 level (figure 3A, online supplemental figure 3A), of which SAHA was chosen for the following experiments since SAHA has been approved for the treatment of cutaneous T-cell lymphoma.^26^ We confirmed that SAHA decreased SOX2 protein levels but not mRNA levels (figure 3B, online supplemental figure 3B). Then, we introduced CHX to inhibit translation of SOX2. The protein level of SOX2 was decreased significantly when cells were treated with SAHA for 12 hours (online supplemental figure 3C), indicated that SAHA prompted SOX2 degradation. Furthermore, the SAHA-related inhibition of SOX2 could mostly be blocked by the proteasome inhibitor MG132 and partially by the autophagy inhibitor bafilomycin A1 (online supplemental figure 3D). Besides, SAHA increased SOX2 acetylation and ubiquitylation levels (online supplemental figure 3E, F). These data demonstrated that SAHA facilitated SOX2 acetylation and proteasome degradation.

To test whether SAHA could reverse the SOX2-dependent CD8+ T-cell tolerance, vector or SOX2 OE melanoma cells were treated with SAHA before cocultured with activated peripheral blood T cells. As expected, SAHA sensitized the melanoma cells to T-cell killing and partially reversed SOX2-induced CD8+ T-cell tolerance, indicating that the function of SAHA might be achieved through targeting SOX2 (figure 3C, D).

Next, vector or SOX2 OE B16/F10 cells were injected into C57BL/6 mice and treated with SAHA (figure 3E). The result showed that SAHA could reduce the tumor burden and partially reversed SOX2-induce tumor promotion effect (figure 3F and G). Tumor infiltrating immune cells analysis displayed that SAHA increased CD8+ T cells and CD8+ cytotoxic T cells infiltration, and partially eliminated SOX2-related CD8+ T cells and CD8+ cytotoxic T cells inhibition (figure 3H–J, online supplemental figure 4A–C). Additionally, SAHA had a mild influence on the CD4+ T/effector T cells, NK cells and Treg cells (figure 3K–O). Based on these findings, we concluded that SAHA could overcome SOX2-related CD8+ T cells tolerance in vivo.

### SAHA enhanced the antitumor effect of IFNγ

**Figure 4:**
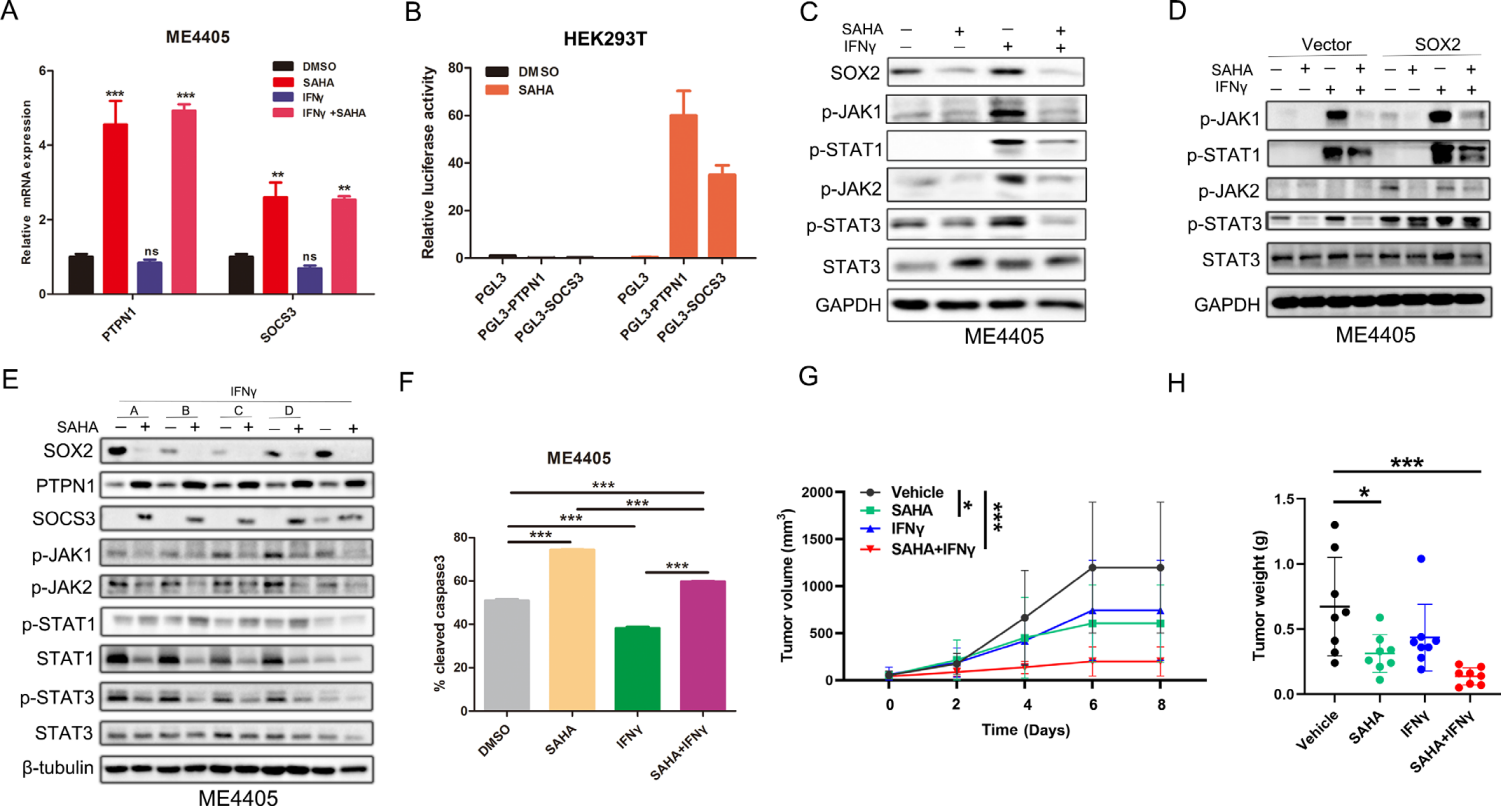
SAHA neutralized SOX2-induced JAK-STAT pathway hyperactivation and augmented the antitumor immunity of IFNγ. (A, B) The expression of ISG.RS mRNA assessed by qPCR assay and luciferase report assay. ME4405 cells were treated with or without 5 µM SAHA for 24 hours. (C, D, E) Change of the p-JAK1/2, p-STAT1/3 level determined by western blot. ME4405 cells (C), Vector or SOX2 OE ME4405 cells (D) were treated with 5 µM SAHA or 1000 IU/mL IFNγ for 24 hours. (E) ME4405 cell were cocultured with SAHA or phosphatase inhibitors (A: Endronate, 1 µM; B: SP112, 1 µM; C: PTP inhibitor, 1 mM; D: PTP1B inhibitor, 50 nM) or 1000 IU/mL IFNγ for 24 hours. (F) Histogram of the percentage of cleaved caspase-3 positive cells determined by flow cytometry. ME4405 cells were pretreated with SAHA or 1000 IU/mL IFNγ for 24 hours and then cocultured with T cells (Tumor: T=10:1). (G, H) Tumor growth curve and xenografts weight of melanoma tumors from C57BL/6 mice (control and IFNγ, n=8; SAHA and combination, n=7). Tumor: T, Tumor cells: T cells. Error bars indicate SEM. One-way ANOVA test. ANOVA, analysis of variance; NS, not significant; ***p<0.001, **p<0.01, *p<0.05.

Mechanistically, SAHA stimulated PTPN1 and SOCS3 transcription (figure 4A and B, (online supplemental figure 5A) and declined the JAK-STAT pathway activity (figure 4C, online supplemental figure 5B). Besides, the p-JAK1/2 and p-STAT1/3 levels were also reduced by SAHA in the SOX2 OE group but were still slightly higher than the vector group (figure 4D, online supplemental figure 5C), confirming that SAHA inhibited JAK-STAT activity through targeting SOX2. To test whether PTPN1 and SOCS3 were required, we treated melanoma cells with SAHA and phosphatase inhibitors for 24 hours. We found that phosphatase inhibitors increased the p-JAK1/2, p-STAT1/3 levels, corroborating that PTPN1 and SOCS3 inhibition could augment the JAK-STAT pathway activity. On PTPN1 and SOCS3 inhibition, SAHA could not fully decrease p-JAK1/2, p-STAT1/3 (figure 4E), suggesting that SAHA blocked SOX2-mediated JAK-STAT pathway excessive activation through stimulating PTPN1 and SOCS3 expression.

Next, we tested whether SAHA could enhance the antitumor effect of IFNγ. SAHA and IFNγ pretreated melanoma cells were cocultured with activated human peripheral blood T cells. We found that SAHA could sensitize the melanoma cells to T-cell killing in IFNγ pretreated group (figure 4F, online supplemental figure 5D). Then, we detected the antitumor effect of SAHA and IFNγ in immune-competent (C57BL/6) mice and immune-compromised (nude) mice. SAHA or IFNγ alone showed mild antitumor response but remarkable tumor regression was achieved in the combination group (figure 4G, H, (online supplemental figure 5E). Besides, the tumors in immunocompromised mice failed to show response to both therapies (online supplemental figure 5F–H), signifying that SAHA could significantly augment the antitumor effect of IFNγ in an immunocompetent context with intact T-cell immunity.

### SAHA reinforced the effect of anti-PD-1

**Figure 5:**
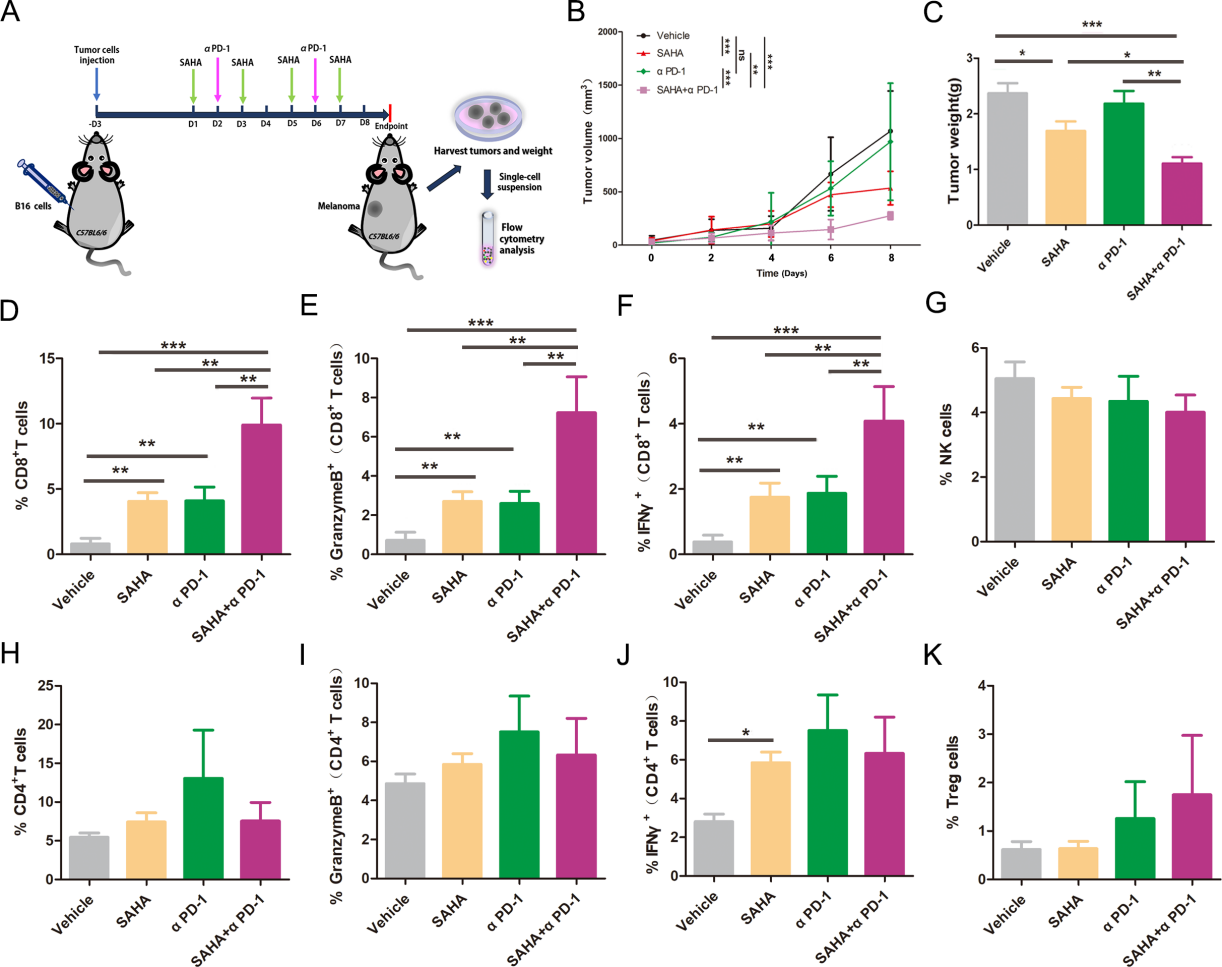
SAHA enhanced CD8+ T function and augmented the effect anti-PD-1. (A) The in vivo treatment schedules for anti-PD-1 and SAHA. (B, C) Tumor growth curves and xenografts weight from C57BL/6 mice (control and anti-PD-1, n=8; SAHA and combination, n=8). (D, G, H, K) Percentage of CD8+ T, NK, CD4+ T and Treg T cells in TILs for each group. (E, F, I, J) Percentage cytotoxic CD8+ T in total CD8+ T cells and cytotoxic CD4+ T in total CD4+ T cells. Error bars indicate SEM. One-way ANOVA test. ANOVA, analysis of variance; NS, not significant; TILs, tumor infiltrating lymphocytes; ^***^p<0.001, ^**^p<0.01, ^*^p<0.05.

Given that SAHA decreased SOX2 expression and recovered the sensitivity of melanoma cells to T-cell killing, we hypothesized that SAHA could enhance the effect of anti-PD-1 in vivo. For this purpose, C57BL/6 mice were inoculated with B16/F10 cells and then treated with SAHA and anti-PD-1 (figure 5A). SAHA demonstrated slight antitumor activity and anti-PD-1 had no antitumor effect, while the combined treatment could significantly decrease tumor growth (figure 5B, C). Then, tumors infiltrating immune cells were detected. CD8+ T cells and CD8+ IFNγ+ T cells or CD8+ GranzymeB+ T cells were slightly increased in SAHA or anti-PD-1 group, but were significantly infiltrated in the combination group (figure 5D–F, online supplemental figure 6A–C). Additionally, neither treatment increased the CD4+ T/effector T-cells, NK cells and Treg cells population (figure 5G–K). Thus, we concluded that SAHA could reinforce the effect of anti-PD1 through overcoming SOX2-ralated CD8+ T-cell tolerance.

### Prognostic impact of SOX2 in anti-PD-1-treated melanoma patients

As our results uncovered that SOX2 caused immune evasion to CD8+ T cells killing through regulating IFNγ pathway, we hypothesized that SOX2 might contribute to the apparent discrepancy of response and OS to anti-PD-1 in CD8+ T-cell inflamed patients. To elucidate it, 84 metastatic melanoma patients with transcriptional profiles and clinical data from the GEO repository were used. 58 patients received nivolumab, 43 had paired prenivolumab (before treatment) and onnivolumab (4 weeks after the initiation of treatment) data, seven had only prenivolumab data and eight had only on-nivolumab data. 26 patients treated with pembrolizumab. Of all the patients treated with anti-PD-1 antibodies, the median patient overall OS was 94.6 (95% CI: 61.7 to 127.5) weeks and consisted of 2 M0, 12 M1a, 13 M1b, 47 M1c and 10 patients with unknown stage. As expected, we found that SOX2 high status indicated a poor OS (p value, prenivolumab: 0.042; onnivolumab: 0.007; prepembrolizumab: 0.025) and PFS (p value, prepembrolizumab: 0.064) in CD8+ T-cell inflamed patients while it could not stratify survival in CD8+ T-cell absent patients (online supplemental figure 7A, C. Similarly, SOX2 also had prognostic effect in IFNγ proficient patients (online supplemental figure 7B).

**Figure 6:**
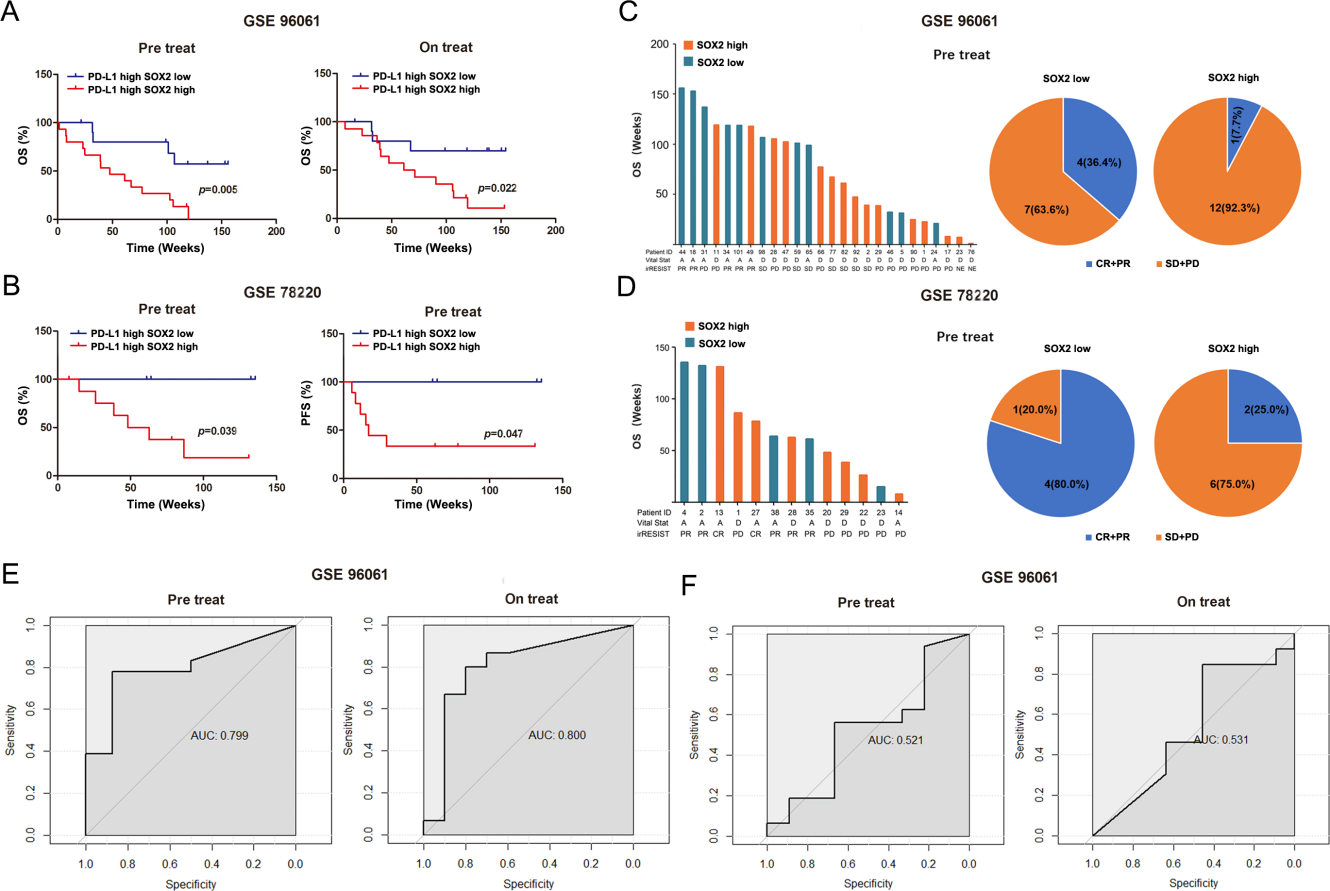
Correlation between SOX2 status and clinical response to anti-PD-1 in PD-L1 high patients. (A, B) Kaplan-Meier survival curves comparing the OS and PFS between SOX2 high and low group in melanoma with PD-L1 high expression; (C, D) Left: Histogram representing the clinical benefit of anti-PD-1 based on SOX2 expression in melanoma with PD-L1 high expression. Right: Pie chart of the proportion of response for each group. (E, F) ROC curves of the prediction model derived from PD-L1high (E) or PD-L1 low (F) melanoma treated with nivolumab therapy. A, alive; AUC, the area under ROC curve; CR, complete response; D, dead. irRECIST, Immune-related Response Evaluation Criteria in Solid Tumors; PFS, progression-free survival; PD, progressive disease; PR, partial response; OS, overall survival; ROC, receiver operating characteristic. Unpaired two-tailed t tests.

Since PD-L1 is widely used to predict clinical outcomes of anti-PD-1 and was strongly associated with CD8+ T infiltration,^27^ we evaluated the prognostic impact of SOX2 across different PD-L1 status. No significant difference between the clinicopathological characteristics of the PD-L1^low^ SOX2^low^, PD-L1^low^ SOX2^high^, PD-L1^high^ SOX2^low^ and PD-L1^high^ SOX2^high^ patients were observed (online supplemental tables 1,2). SOX2 high status (both prenivolumab and onnivolumab) corresponded to a poor OS in PD-L1 high patients (p value, prenivolumab: 0.005; onnivolumab: 0.022) (figure 6A) but it could not stratify the survival of PD-L1 low patients (p value, prenivolumab: 0.826; onnivolumab: 0.863) (online supplemental figure 8A). These results indicated that the baseline and evolution status of SOX2 had a prognostic value for high PD-L1 patients regardless of the intratumor heterogeneity evolution during the nivolumab treatment.^16^ Similarly, for patients treated with pembroluzimab, SOX2 was also correlated with poor OS and PFS (p value, OS: 0.039; PFS: 0.047) in PD-L1 high patients (figure 6B) while it could not stratify the survival of PD-L1 low patients (p value, OS: 0.589; PFS: 0.854) (online supplemental figure 8B).

Besides, ORR was higher in PD-L1 high patients with SOX2 low expression than SOX2 high expression (SOX2 low vs SOX2 high, prenivolumab: 36.4% (4 of 11) vs 7.7% (1 of 13); onnivolumab: 27.3% (3 of 11) vs 15.4% (2 of 13) (figure 6C, online supplemental figure 8C); prepembroluzimab: 80.0% (4 of 5) vs 25.0% (2 of 8) (figure 6D). Multivariate cox regression analysis using BRAF/NRAS/NF1 mutation status, tumor stage and previous ipilimumab treatment as adjusting factors identified SOX2 as a negative independent predictor for OS in PD-L1 high patients (prenivolumab, HR: 5.67, 95% CI: 1.68 to 19.17, p value: 0.005; onnivolumab, HR: 3.69, 95% CI: 1.02 to 13.40, p value: 0.047) (online supplemental tables 3,4). According to the AUC of the ROC model, we found that the SOX2 status was a better predictor in high PD-L1 patients than low PD-L1 patients (AUC, PD-L1 high vs PD-L1 low, prenivolumab: 0.799 vs 0.521; onnivolumab: 0.800 vs 0.531) (figure 6E and F). The patients treated with pembroluzimab were not included in the analysis due to small sample size (26 patients). We found that SOX2 was an independent predictor for poor survival and resistance to anti-PD-1 therapy in melanoma with high PD-L1 expression.

## Discussion

In this study, we found that SOX2 promoted immune evasion to CD8+ T-cell killing through inhibiting of PTPN1 and SOCS3 transcription, causing the hyperactivation of the JAK-STAT pathway and overexpression of ISG.RS. SAHA unleashed SOX2-related CD8+ T-cell tolerance and enhanced antitumor effect of anti-PD1. Besides, SOX2 could predict a poor survival to ICIs in melanoma with PD-L1 high expression, thus providing a further mechanistic rationale for combining SAHA with ICIs.

PD-L1 high expression with abundant T-cell or IFNγ signaling indicated an immunogenic state, PD-L1 low expression with absent T cells or IFNγ signaling indicated immune ignorance state.^4 28 29^ Usually, immunogenic state is often associated with prolong survival and response to ICIs. However, immunogenic tumors with pre-existing CD8+ T cells were tolerant and could not be unleashed by ICIs. A great deal of researches have underlined that bystander T cells recognizing cancer-unrelated epitopes, the presence of suppressive immune cells, insensitivity to interferons or metabolite and cytokine dysregulation in the TME contributed to resistance of ICIs.^30 31^ Duration IFNγ signaling was also reported to augments adaptive ICIs resistance through inducing overexpression of ISG.RS.^20 21^ Findings from our experiments showed that SOX2 account for the immune evasion through alleviating the IFNγ signaling and ISG.RS expression. IFNγ is the most potent driver to induce PDL1 expression.^21 29^ These may help to explain why SOX2 had prognostic value in melanoma with PD-L1 expression, which indicting an immunogenic state.

We found SOX2 KD also decreased the IFNγ-induced PD-L1 expression, which was opposite from the traditional opinion that PD-L1 favor a better outcome to anti-PD1. Xu *et al* mentioned that OE of PD-L1 induced by IFN-γ could limit the effect of ICIs.^32^ Additionally, PD-L1 blockade inside tumors was not sufficient to mediate regression, as PD-L1 signaling in defined antigen-presenting cells also inhibited T-cell activation.^33^ Targeting SOX2 could suppress the JAK-STAT pathway and block the IFNγ-induced ISG.RS OE (including PD-L1) in tumor cells, thus overcome the IFNγ-related resistant to CD8+ T-cell killing and potentiated the effect of anti-PD-1. We supposed that the “induced” PD-L1 low expression as a result of SOX2 inhibition in immunogenic patients indicated decreasing expression of ISG.RS and thereby unleashing the IFNγ induced therapeutic resistance, which was different from “primary” PD-L1 low expression in immune ignorance patients.

Numerous biomarkers, predominantly involving indices from the tumor cells (gene expression profile and tumor mutation load) or cells from the microenvironment (tumor-infiltrating lymphocytes) and blood (circulating cells, cytokines/chemokines, and exosomes), are also recognized to predict clinical outcomes and identifying subgroups of patients who will benefit from treatments.^34–36^ Of them, PD-L1 is a widely used biomarker in clinical practice and often correlate with favor clinical response, but nearly half of the PD-L1 expression patients do not achieve ORR.^37 38^ Till present, the implementation of any of these biomarkers is challenging. Our results showed that SOX2 low expression conferred significant improvement in OS and PFS, and was an independent predictor of OS in melanoma with PD-L1 high expression. This was an encouraging improvement of predictive value over PD-L1 alone. However, findings from our multivariate analyzes were significantly limited by the small numbers in each group.

Previous reports showed that SOX2 enhanced the activities of tyrosine kinases p-STAT3,^39^ p-ERK^40^ and p-Src^41^ but the mechanisms remained largely unknown. We found that SOX2 induced phosphatases PTPN1 and SOCS3 expression, which were reported to dephosphorylate and counter the activities of tyrosine kinases. Recent studies revealed that phosphatases had a controversial in antitumor immune response: PTEN induced a tumor-intrinsic T-cell-inflamed state resulting in response to ICIs^42^ and SOCS2 impaired the dendritic cell-based priming of T cells and limited adaptive antitumor immunity.^43^ Here, we uncovered that tumor intrinsic PTPN1 and SOCS3 could promote antitumor immunity through restrain the activity of JAK-STAT pathway. Several clinical trials (NCT02646748 and NCT03012230) employing the JAK inhibitors in combination anti-PD-1 are underway, but early results have not been favorable.^44^ SOX2 is overexpressed in melanoma,^45^ genetic perturbation of SOX2 could promote the expression of PTPN1 and SOCS3 and feedback inhibition of JAK-STAT pathway, making SOX2 a promising target to control aberrant JAK-STAT activity.

SOX2 is an “undrugable” transcription factor, but methylation and acetylation could promote it degradation.^24 25^ Based on this, we conducted an epigenetic compounds library screen and identified that SAHA, the Food and Drug Administration (FDA) approved inhibitor, decreased SOX2 level and potentiated the antitumor immunity of ICIs. Clinical studies combining SAHA with ICIs are ongoing (NCT02638090, NCT02538510, NCT02619253, NCT02395627), the most crucial SAHA-controlled mechanisms and biomarkers to discriminate priority patients who will and will not respond remained to be defined.^46 47^ Preclinical studies suggest that the mechanisms might be attributed to the expression of immune-related genes and tumor antigens.^48^ This study uncovered that SAHA increased the acetylation and proteasome degradation of SOX2, thus relieving the SOX2-related inhibitory function on T-cell antimelanoma immunity. Collectively, we identified SOX2 as a biomarker for identifying melanoma patients who will response to the combination therapies of SAHA and ICIs, while its predictive value of response or survival warrants further investigation in prospective clinical trials.

Efforts have been made to improve the therapeutic efficacy of patients absent with PD-L1 expression. In contrast, our findings manifested significant implications for combined therapies in melanoma with high PD-L1 expression. We found that SOX2 was a biomarker of poor clinical response in PD-L1 high expressing patients and the inhibition of SOX2 by SAHA significantly enhanced the therapeutic efficacy of ICIs, indicating SOX2 as a potential therapeutic target in combination therapy.

## Conclusions

Understanding additional mechanisms accounting for resistance to ICIs in melanoma patients with PD-L1 high expression would tailor more precise and effective combinational therapeutics. This study identified that SOX2 could lead to immune evasion of CD8+ T-cell killing by decreasing phosphatase PTPN1 and SOCS3 transcription, resulting in duration activation of the JAK-STAT pathway and thereby OE of ISG.RS. SAHA could promote SOX2 degradation and argument the therapeutic effect of anti-PD1 therapy. These findings highlighted a SOX2-dependent resistant mechanism to anti-PD1 therapy in melanoma with PD-L1 high expression, thus proposing a new biomarker and combination therapeutic paradigm for treating such patients.

10.1136/jitc-2020-001037.supp2 Supplementary data
