# Supplementary material for: SOX2 promotes resistance of melanoma with PD-L1 high expression to T-cell-mediated cytotoxicity that can be reversed by SAHA
Source: J Immunother Cancer. 2020 Nov 6;8(2):e001037. doi: 10.1136/jitc-2020-001037 (PMC7651737; doi:10.1136/jitc-2020-001037)
Supplement: Supplementary data [file jitc-2020-001037supp001.pdf]

# Supplementary Materials

| |
|---|
| **Supplementary figure 1.** The impact of SOX2 on $CD8^+$ T cell killing *in vivo*. |
| **Supplementary figure 2.** The function of SOX2 on tumor growth and patients survival. |
| **Supplementary figure 3.** SAHA decreased SOX2 level through promoting SOX2 acetylation and degradation. |
| **Supplementary figure 4.** Representative dot plots of indicated cells. |
| **Supplementary figure 5.** SAHA induced PTPN1 and SOCS3 expression and recovered the sensitivity of melanoma cells to T cell killing. |
| **Supplementary figure 6.** Representative dot plots of indicated cells. |
| **Supplementary figure 7.** The correlation between SOX2 and clinical response to anti-PD-1 based on CD8 or IFNγ expression. |
| **Supplementary figure 8.** The correlation between SOX2 and clinical response to anti-PD-1 in melanoma with PD-L1 low expression. |
| **Supplementary table 1.** Comparison of clinicopathologic characteristics based on SOX2 and PD-L1 status before nivolumab therapy. |
| **Supplementary table 2.** Comparison of clinicopathological characteristics based on SOX2 and PD-L1 status 4 weeks after initiation of nivolumab. |
| **Supplementary table 3.** Multivariate Cox regression analysis of clinicopathological factors overall survival in melanoma patients before nivolumab therapy. |
| **Supplementary table 4.** Multivariate Cox regression analysis of clinicopathologic factors overall survival in melanoma patients 4 weeks after initiation of nivolumab. |

**Supplementary figure 1. The impact of SOX2 on CD8$^+$ T cell killing *in vivo*.**

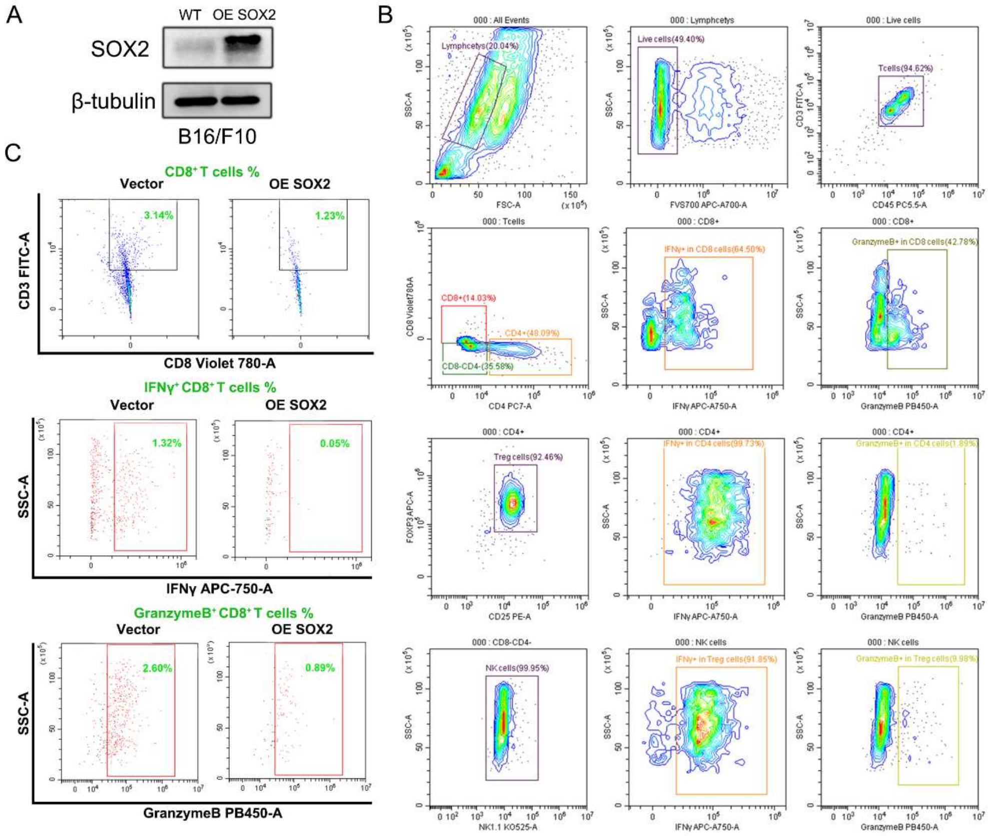


(A)The expression of SOX2 was assessed with western blot analysis. B16/F10 cells transfected with control vector or plasmids encoding Flag-mSOX2. (B) Gating strategy for multicolor flow cytometry analysis of tumor infiltrating lymphocytes. (C) Representative dot plots of CD8$^+$ T in the TILs, CD8$^+$ IFNγ$^+$ T in CD8$^+$ T cells and CD8$^+$ GranzymeB$^+$ T in CD8$^+$ T cells isolated from a representative mouse for each group.

**Supplementary figure 2. The function of SOX2 on tumor growth and patients survival.**

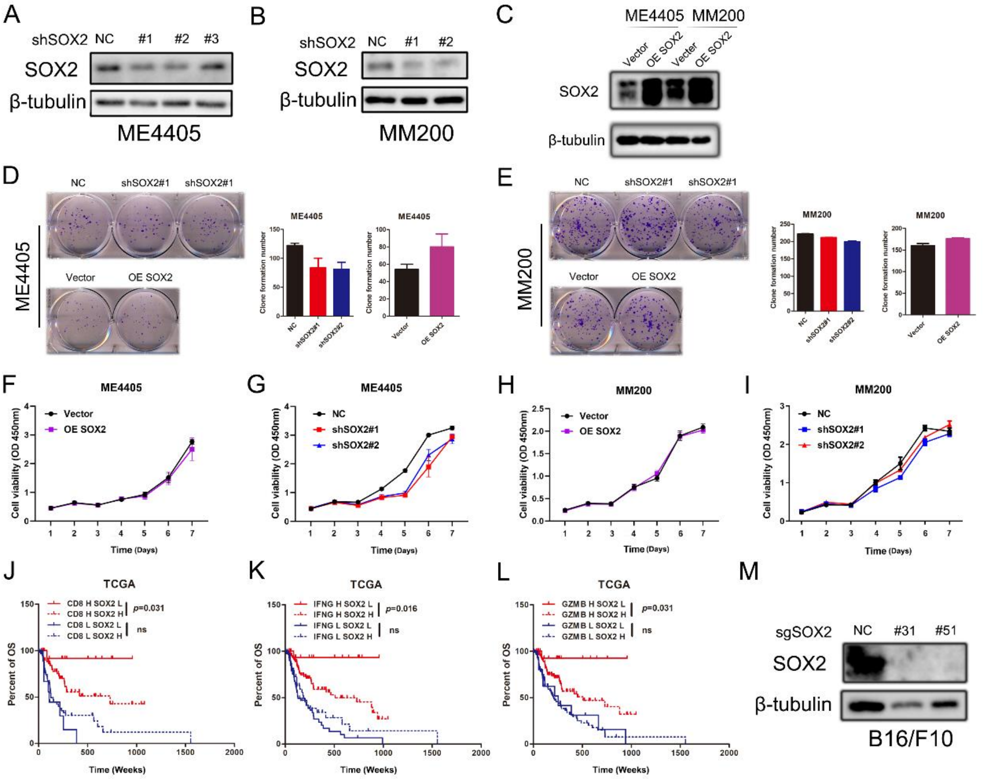


(A-C) The expression of SOX2 was assessed with western blot analysis. ME4405 cells (A) and MM200 cells (B) transfected with negative control or plasmids encoding shSOX2; ME4405 cells and MM200 cells were transiently transfected with control vector or plasmids encoding Flag-hSOX2 (C). (D-I) The function of SOX2 on tumor growth *in vitro*. ME4405 cells and MM200 cells transfected with indicated plasmid and sent for colony formation assay (D, E) and CCK8 assay (F-I). (J-L) Kaplan-Meier survival curves comparing the OS based on SOX2 and CD8 status (J), SOX2 and IFNG status(K), SOX2 and GZMB status(L). (M) The expression of SOX2 in B16/F10 cells transfected with indicated negative control or plasmids encoding shSOX2. Error bars indicate SEM. Unpaired two-tailed t tests for two groups comparison. One-way ANOVA test for three or more groups comparison. NS, not significant; $^{***}$ $p$ <0.001, $^{**}$ $p$ <0.01, $^{*}$ $p$ <0.05.

**Supplementary figure 3. SAHA decreased SOX2 level through promoting SOX2**

**acetylation and degradation.**

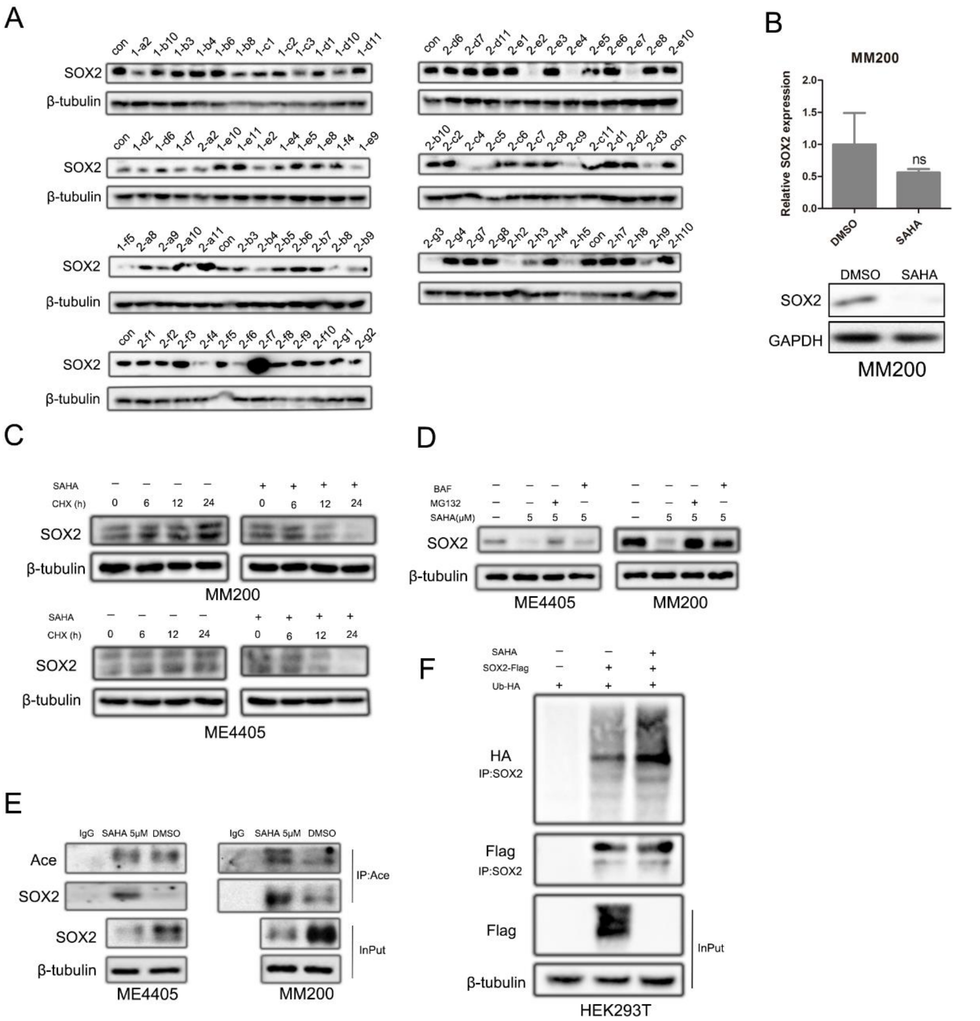


(A) Screen results by western blot in MM200 cells. MM200 cells were treated with 1000 IU/mL IFNγ and 5μM epigenetic inhibitors for 24 hr. (B) Change of SOX2 protein level and mRNA according to western blot and qPCR assay. MM200 cells were treated with or without 5μM SAHA for 24hr. (C) Change of SOX2 protein level determined by western blot. Melanoma cells were treated with 25 μg/ml CHX alone or 25 μg/ml CHX plus 5 μM SAHA for different time. (D) SAHA promoted SOX2 degradation. Melanoma cells were incubated with 5 μM SAHA for 24hr, and then treated with10 μM MG132 for 6hr or 100 nM Bafilomycin A1 for 12hr. (E) SAHA increased the acetylation SOX2. Melanoma cells were treated with SAHA for 24hr. Immunoprecipitated acetylation was subjected to anti-SOX2 western blotting. (F)

SAHA increased ubiquitination of endogenous SOX2. HEK293T cells were transiently co-transfected with vector and plasmids encoding Flag-hSOX2 or HA-Ub, treated with SAHA for 24hr. Immunoprecipitated Flag-hSOX2 was subjected to anti-HA-Ub western blotting. Error bars indicate SEM. Unpaired two-tailed t test, NS, not significant.

**Supplementary figure 4. Representative dot plots of indicated cells.**

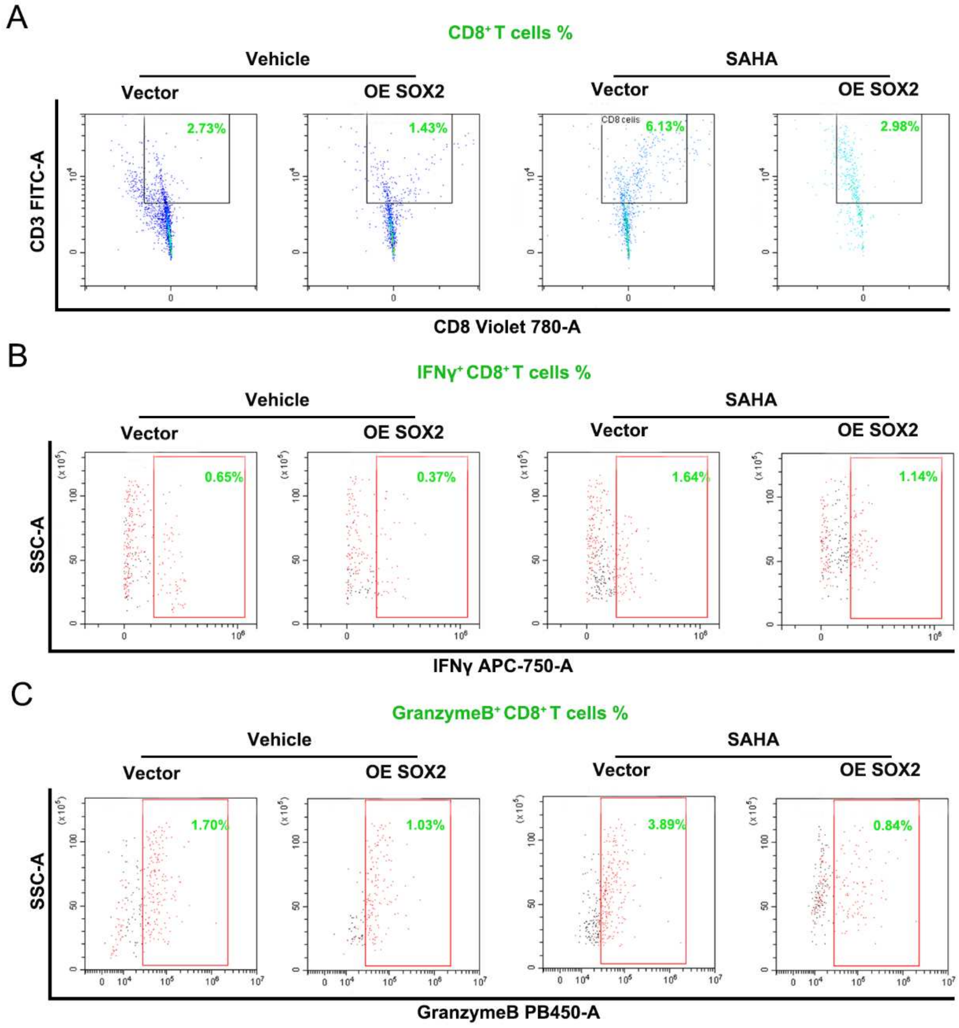


(A-C) Representative dot plots of $CD8^+$ T in the TILs (A), $CD8^+$ $IFN\gamma^+$ T in $CD8^+$ T cells (B) and $CD8^+$ $GranzymeB^+$ T in $CD8^+$ T cells (C) isolated from a representative mouse for each group.

**Supplementary figure 5. SAHA induced PTPN1 and SOCS3 expression and**

**recovered the sensitivity of melanoma cells to T cell killing.**

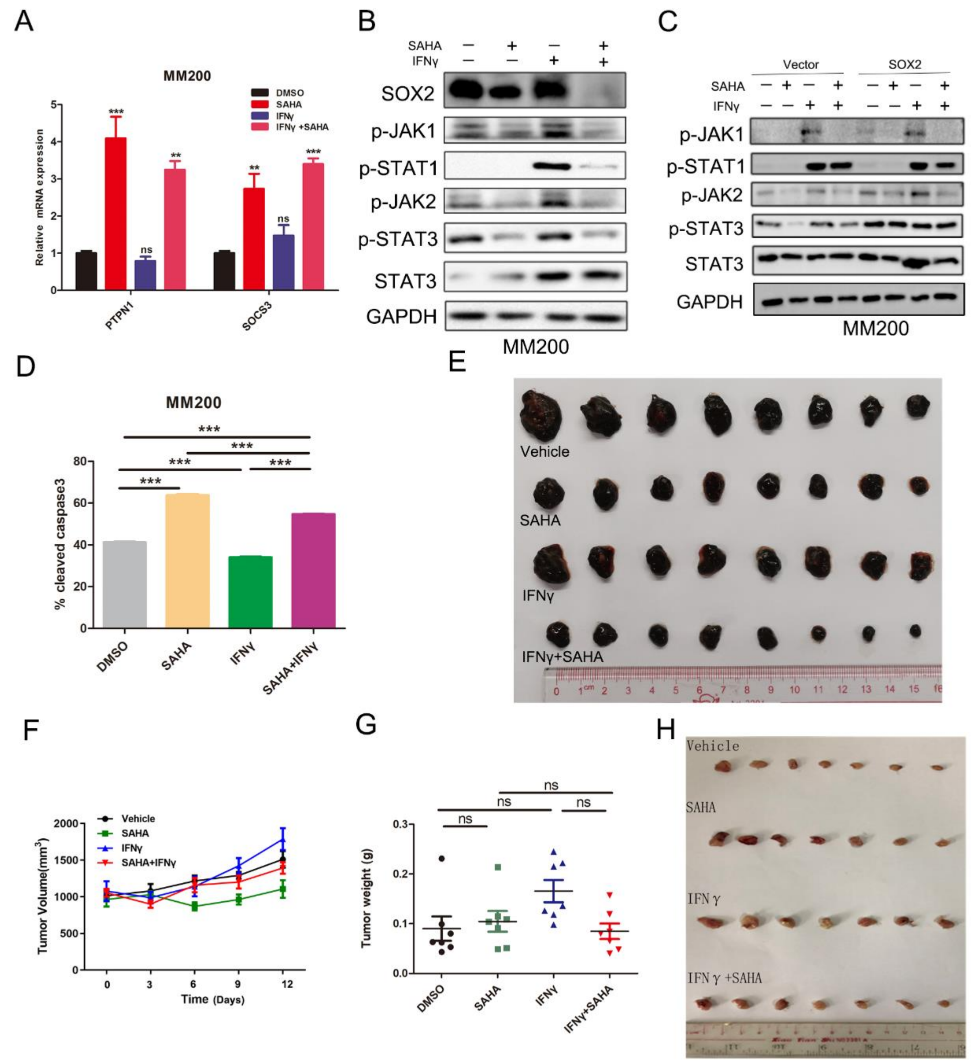


(A) PTNP1 and SOCS3 mRNA level according to qPCR. MM200 cells were treated with 5μM SAHA and 1000 IU/mL IFNγ for 24 hr. (B, C) Change of the p-JAK1/2, p-STAT1/3 level determined by western blot. MM200 cells (B), vector or SOX2 OE MM200 cells (C) were treated with 5μM SAHA or 1000 IU/mL IFNγ for 24hr. (D) Histogram of the percentage of cleaved caspase-3 positive cells determined by flow cytometry. MM200 cells were pretreated with SAHA or 1000 IU/mL IFNγ for 24hr and then co-cultured with T cells for 6-8hr (Tumor: T= 10:1). (F, G) Tumor growth curve and xenografts weight of nude mice. (E, H) The picture of tumors in C57BL/6 mice and nude mice. Error bars indicate SEM. One-way ANOVA test, NS, not

significant; $^{***}p$ <0.001, $^{**}p$ <0.01, $^{*}p$ <0.05.

**Supplementary figure 6. Representative dot plots.**

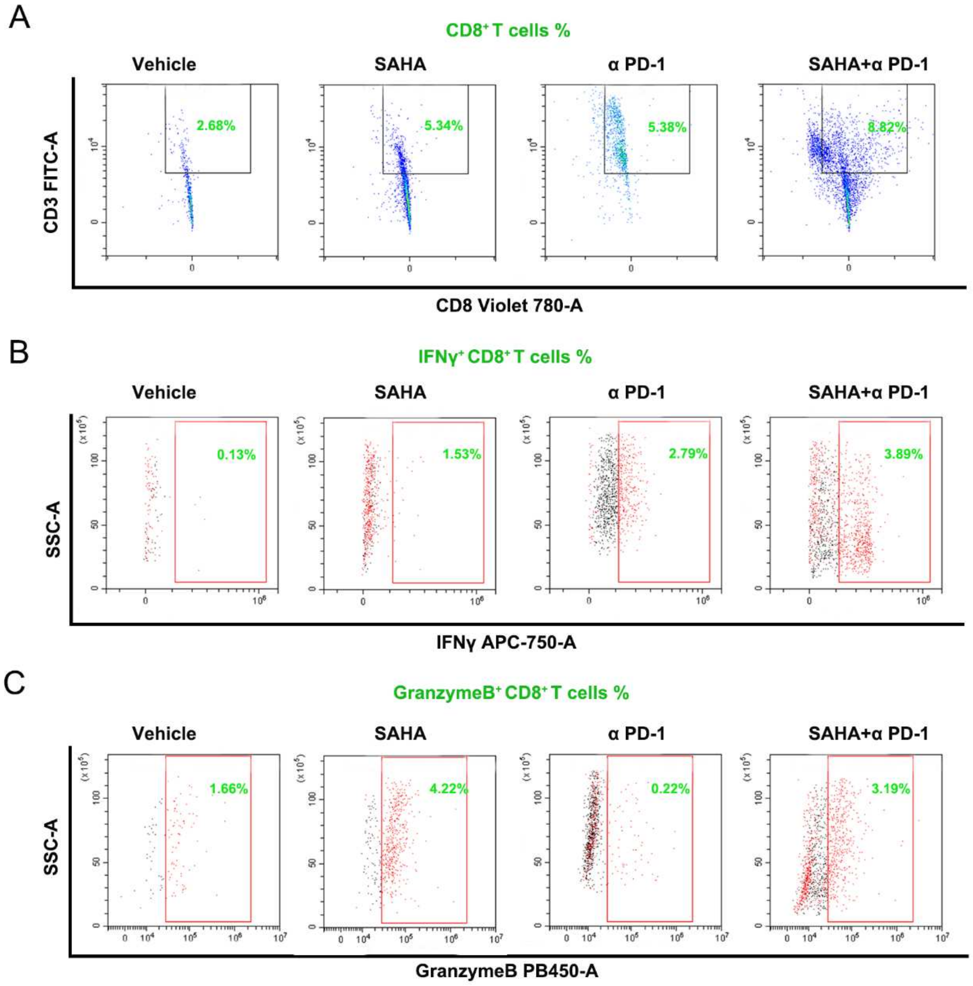


(A-C) Representative dot plots of CD8$^+$ T in the TILs (A), CD8$^+$ IFNγ$^+$ T in CD8$^+$ T cells (B) and CD8$^+$ GranzymeB$^+$ T in CD8$^+$ T cells (C) isolated from a representative mouse for each group.

**Supplementary figure 7. The correlation between SOX2 and clinical response to anti-PD-1 based on CD8 or IFNγ expression.**

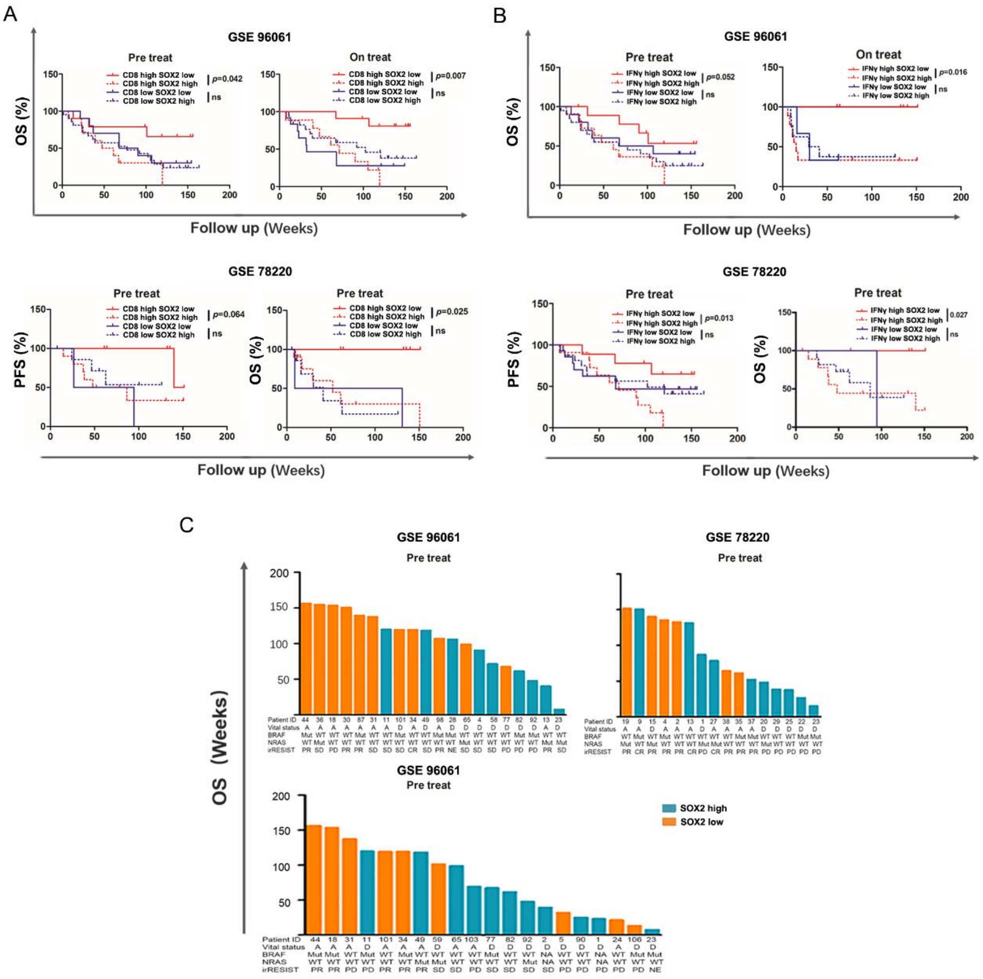


(A) Kaplan-Meier survival curves comparing the OS and PFS based on SOX2 and CD8 status. (B) Kaplan-Meier survival curves comparing the OS and PFS based on SOX2 and IFNγ status. (C) Histogram representing the clinical benefit of anti-PD-1 based on SOX2 expression in CD8 high patients. A, alive; D, dead. irRECIST, Immune-related Response Evaluation Criteria in Solid Tumors; CR, complete response; PR, partial response; PD, progressive disease. NS, not significant.

**Supplementary figure 8. The correlation between SOX2 and clinical response to anti-PD-1 in melanoma with PD-L1 low expression.**

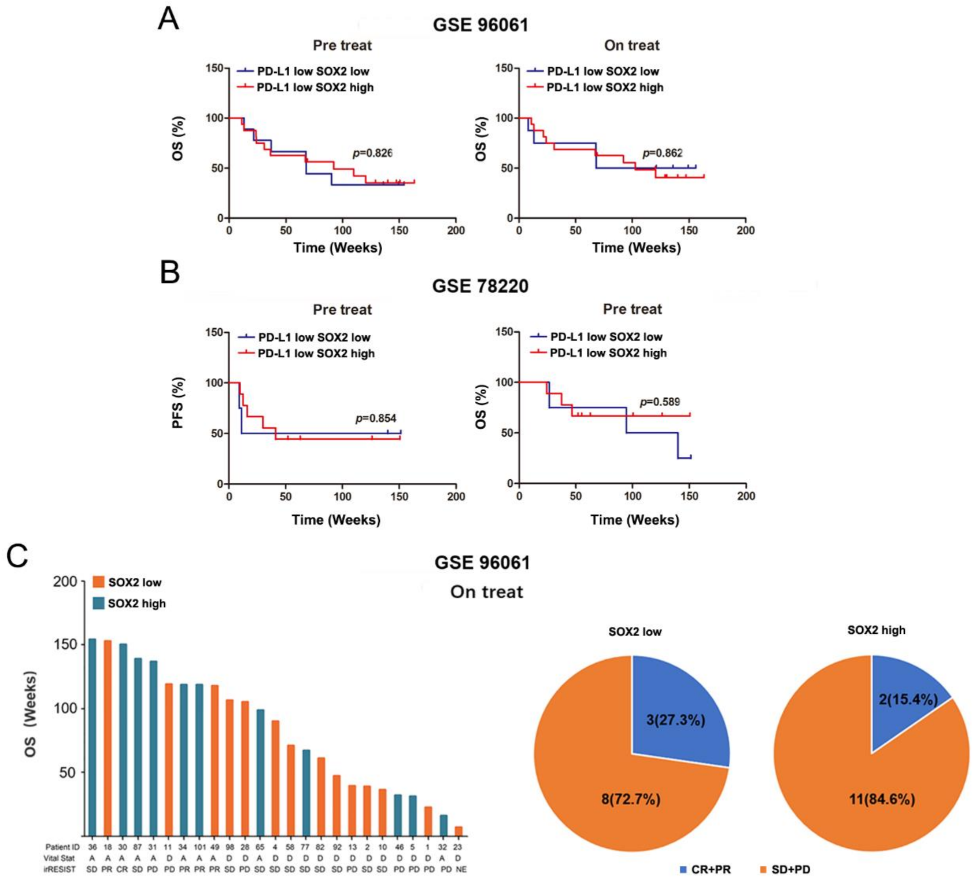


(A, B) Kaplan-Meier survival curves comparing the OS and PFS between the SOX2 high and low group in melanoma with PD-L1 low expression; (C) Left: Histogram representing the clinical benefit of anti-PD-1 based on SOX2 expression in melanoma with PD-L1 low expression. Right: Pie chart of the proportion of response for each group. A, alive; D, dead. irRECIST, Immune-related Response Evaluation Criteria in Solid Tumors; CR, complete response; PR, partial response; PD, progressive.

**Supplementary table 1**. Comparison of the clinicopathological characteristics based on SOX2 and PD-L1 status before nivolumab therapy.

| | PD-L1 low tumor (< 50%) | | PD-L1 high tumor (> 50%) | | |
|---|---|---|---|---|---|
| **Characteristics** | **SOX2 L[1]** | **SOX2 H[2]** | **SOX2 L** | **SOX2 H** | *p* |

BMJ Publishing Group Limited (BMJ) disclaims all liability and responsibility arising from any reliance placed on this supplemental material which has been supplied by the author(s)


| | (n =9) | (n =16) | *p* | (n =11) | (n =15) | *p* | |
|---|---|---|---|---|---|---|---|
| **Primary site** | | | 0.671 | | | 0.228 | 1.000 |
| Cutaneous | 5 (55.6) | 11 (68.8) | | 5 (45.5) | 11 (73.3) | | |
| Other | 4 (44.4) | 5 (31.2) | | 6 (54.5) | 4 (26.7) | | |
| **Mutation** | | | 0.397 | | | 0.228 | 1.000 |
| Triple Wt[3, 4] | 5 (55.6) | 5 (31.3) | | 6 (54.5) | 4 (26.7) | | |
| Other | 4 (44.4) | 11 (68.8) | | 5 (45.5) | 11 (73.3) | | |
| **Stage** | | | 0.411 | | | 0.428 | 0.404 |
| M1c[5] | 6 (66.7) | 7 (43.8) | | 3 (27.3) | 7 (46.7) | | |
| Other | 3 (33.3) | 9 (56.2) | | 8 (72.7) | 8 (53.3) | | |
| **Previous IPI[6]** | | | 1.000 | | | 1.000 | 1.000 |
| Progress | 5 (55.6) | 8 (50.0) | | 5 (45.5) | 8 (53.3) | | |
| Naive | 4 (44.4) | 8 (50.0) | | 6 (54.5) | 7 (46.7) | | |

1. L, Low expression; 2. H, High expression; 3. Wt, Wild type; 4. Triple Wt, without mutation of BRAF, NRAS and NRF2; 5. M1c, The stage of patients with melanoma is M1c, according to standards in the AJCC of 2018; 6. IPI, International Prognosis Index.

**Supplementary table 2.** Comparison of the clinicopathological characteristics based on SOX2 and PD-L1 status 4 weeks after initiation of nivolumab.

| | **PD-L1 low tumor (< 50%)** | | | **PD-L1 high tumor (> 50%)** | | | |
|---|---|---|---|---|---|---|---|
| **Characteristics** | **SOX2 L[1]** | **SOX2 H[2]** | | **SOX2 L** | **SOX2 H** | | *p* |
| | (n =8) | (n =16) | *p* | (n =11) | (n =14) | *p* | |

| | | | | | | | |
|---|---|---|---|---|---|---|---|
| **Primary site** | | | 1.000 | | | 0.656 | 0.762 |
| Cutaneous | 5 (62.5) | 11 (68.8) | | 7 (63.6) | 11 (78.6) | | |
| Other | 3 (37.5) | 5 (31.2) | | 4 (36.4) | 3 (21.4) | | |
| **Mutation** | | | 1.000 | | | 0.115 | 0.567 |
| Triple Wt[3, 4] | 4 (50.0) | 7 (43.8) | | 6 (54.5) | 3 (21.4) | | |
| Other | 4 (50) | 9 (56.3) | | 5 (45.5) | 11 (78.6) | | |
| **Stage** | | | 0.211 | | | 1.000 | 0.154 |
| M1c[5] | 6 (75.0) | 7 (43.8) | | 3 (27.3) | 5 (35.7) | | |
| Other | 2 (25.0) | 9 (56.2) | | 8 (72.7) | 9 (64.3) | | |
| **Previous IPI[6]** | | | 0.667 | | | 1.000 | 0.387 |
| Progress | 6 (75.0) | 10 (62.5) | | 6 (54.5) | 7 (50.0) | | |
| Naive | 2 (25.0) | 6 (37.5) | | 5 (45.5) | 7 (50.0) | | |

1. L, Low expression; 2. H, High expression; 3. Wt, Wild type; 4. Triple Wt, without mutation of BRAF, NRAS and NRF2; 5. M1c, The stage of patients with melanoma is M1c, according to standards in the AJCC of 2018; 6. IPI, International Prognosis Index.

**Supplementary table 3**. Multivariate Cox regression analysis of clinicopathological factors for overall survival in melanoma patients before nivolumab therapy.

| | **Univariate Cox** | | | | | | | | **Multivariate Cox** | | | | | | | |
|---|---|---|---|---|---|---|---|---|---|---|---|---|---|---|---|---|
| | **PD-L1 low tumor** | | | | **PD-L1 high tumor** | | | | **PD-L1 low tumor** | | | | **PD-L1 high tumor** | | | |
| | HR[1] | 95% CI[2] | | *p* | HR | 95% CI | | *p* | HR | 95% CI | | *p* | HR | 95% CI | | *p* |
| | | Lower | Upper | | | Lower | Upper | | | Lower | Upper | | | Lower | Upper | |
| **Priamray site** | | | | 0.51 | | | | 0.760 | | | | | | | | |
| Cutaneous | | | | | | | | | | | | | | | | |
| Other | 1.41 | 0.51 | 3.91 | | 0.86 | 0.32 | 2.30 | | | | | | | | | |
| **Mutation** | | | | 0.26 | | | | 0.751 | | | | | | | | |
| TripleWt[3, 4] | | | | | | | | | | | | | | | | |
| Other | 1.83 | 0.63 | 5.28 | | 1.17 | 0.44 | 3.14 | | | | | | | | | |
| **Stage** | | | | 0.25 | | | | 0.000 | | | | | | | | 0.000 |
| M1c[5] | | | | | | | | | | | | | | | | |
| Other | 0.56 | 0.21 | 1.50 | | 0.17 | 0.06 | 0.46 | | | | | | 0.13 | 0.04 | 0.39 | |
| **Previouw IPI[6]** | | | | 0.58 | | | | 0.786 | | | | | | | | |
| Naive | | | | | | | | | | | | | | | | |
| Progress | 0.76 | 0.28 | 2.03 | | 1.14 | 0.44 | 2.96 | | | | | | | | | |
| **SOX2 Level** | | | | 0.86 | | | | 0.010 | | | | | | | | 0.005 |
| Low | | | | | | | | | | | | | | | | |
| High | 0.91 | 0.33 | 2.52 | | 4.42 | 1.43 | 13.73 | | | | | | 5.67 | 1.68 | 19.17 | |

1. HR, Hazard Ratio; 2. CI, Confidence Interval; 3. Wt, Wild type; 4. Triple Wt, without mutation of BRAF, NRAS and NRF2; 5. M1c, The stage of patients with melanoma is M1c, according to standards i the AJCC of 2018; 6. IPI, International Prognosis Index.

**Supplementary table 4**. Multivariate Cox regression analysis of the clinicopathological factors for overall survival in melanoma patients 4 weeks after initiation of nivolumab.

| | Univariate Cox | | | | | | | | Multivariate Cox | | | | | | | |
|---|---|---|---|---|---|---|---|---|---|---|---|---|---|---|---|---|
| | PD-L1 low tumor | | | | PD-L1 high tumor | | | | PD-L1 low tumor | | | | PD-L1 high tumor | | | |
| | HR[1] | 95% CI[2] | | *p* | HR | 95% CI | | *p* | HR | 95% CI | | *p* | HR | 95% CI | | *p* |
| | | Lower | Upper | | | Lower | Upper | | | Lower | Upper | | | Lower | Upper | |
| **Priamray site** | | | | 0.89 | | | | 0.777 | | | | | | | | |
| Cutaneous | | | | | | | | | | | | | | | | |
| Other | 0.92 | 0.28 | 3.00 | | 0.85 | 0.27 | 2.67 | | | | | | | | | |
| **Mutation** | | | | 0.51 | | | | 0.151 | | | | | | | | |
| TripleWt[3, 4] | | | | | | | | | | | | | | | | |
| Other | 1.46 | 0.48 | 4.48 | | 2.32 | 0.74 | 7.33 | | | | | | | | | |
| **Stage** | | | | 0.06 | | | | 0.091 | | | | | | | | 0.160 |
| M1c[5] | | | | | | | | | | | | | | | | |
| Other | 0.31 | 0.10 | 1.03 | | 0.41 | 0.15 | 1.15 | | | | | | 0.47 | 0.16 | 1.35 | |
| **Previouw IPI[6]** | | | | 0.72 | | | | 0.929 | | | | | | | | |
| Naive | | | | | | | | | | | | | | | | |
| Progress | 0.82 | 0.27 | 2.51 | | 0.95 | 0.34 | 2.67 | | | | | | | | | |
| **SOX2 Level** | | | | 0.86 | | | | 0.034 | | | | | | | | 0.047 |
| Low | | | | | | | | | | | | | | | | |
| High | 1.11 | 0.34 | 3.61 | | 3.98 | 1.11 | 14.23 | | | | | | 3.69 | 1.02 | 13.40 | |

1. HR, Hazard Ratio; 2. CI, Confidence Interval; 3. Wt, Wild type; 4. Triple Wt, without mutation of BRAF, NRAS and NRF2; 5. M1c, The stage of patients with melanoma is M1c, according to standards in the AJCC of 2018; 6. IPI, International Prognosis Index.
