# Supplementary material for: SOX2 promotes resistance of melanoma with PD-L1 high expression to T-cell-mediated cytotoxicity that can be reversed by SAHA
Source: J Immunother Cancer. 2020 Nov 6;8(2):e001037. doi: 10.1136/jitc-2020-001037 (PMC7651737; doi:10.1136/jitc-2020-001037)
Supplement: Supplementary data [file jitc-2020-001037supp002.pdf]

SOX2 promotes resistance of melanoma with PD-L1 high expression to T cell-mediated cytotoxicity that can be reversed by SAHA

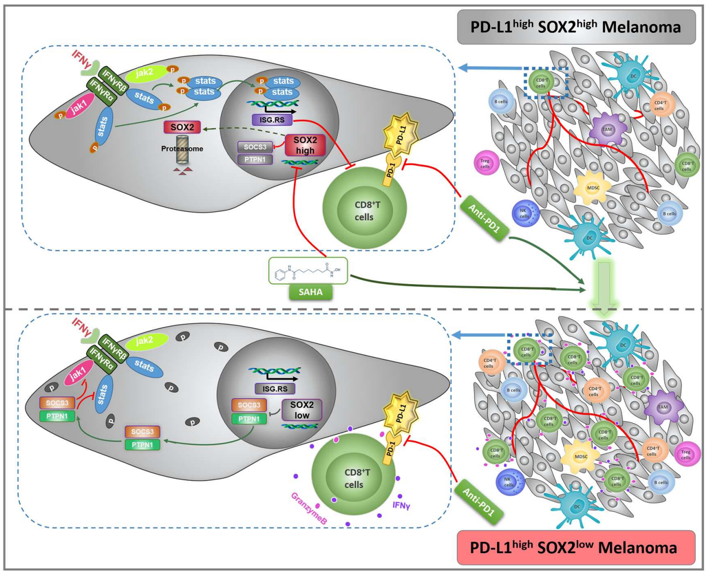

Authors

Rui-Yan Wu, Cai-Qin Wang, Zhi-Ming Li, Jian Xiao, Chun-Yan Li, Xue-Min Wang, Peng-Fei Kong, Jiang-Hua Cao, Fu-Xue Huang, Zhi-Ling Li, Yun Huang, Yu-Hong Chen, Xuan Li, Dong Yang, Hai-Liang Zhang, Jia Mai, Gong-Kan Feng, Rong Deng, Xiao-Feng Zhu

Summary

SOX2 causes immune evasion to T cell-mediated cytotoxicity and resistance to anti-PD-1 in melanoma with PD-L1 high expression.
SOX2 inhibits phosphatase PTPN1 and SOCS3 transcription, which resulted in duration activation of the JAK-STAT pathway and thereby overexpression of ISG.RS.
SAHA promotes SOX2 degradation and argument the therapeutic effect of anti-PD1 therapy.
